# Consensus of gene expression phenotypes and prognostic risk predictors in primary lung adenocarcinoma

**DOI:** 10.18632/oncotarget.10641

**Published:** 2016-07-16

**Authors:** Markus Ringnér, Johan Staaf

**Affiliations:** ^1^ Division of Oncology and Pathology, Department of Clinical Sciences Lund, Lund University, Medicon Village, SE 22381, Lund, Sweden

**Keywords:** lung adenocarcinoma, molecular subtype, gene expression, prognostic, risk predictor

## Abstract

Transcriptional profiling of lung adenocarcinomas has identified numerous gene expression phenotype (GEP) and risk prediction (RP) signatures associated with patient outcome. However, classification agreement between signatures, underlying transcriptional programs, and independent signature validation are less studied. We classified 2395 transcriptional adenocarcinoma profiles, assembled from 17 public cohorts, using 11 GEP and seven RP signatures, finding that 16 signatures were associated with patient survival in the total cohort and in multiple individual cohorts. For significant signatures, total cohort hazard ratios were ~2 in univariate analyses (mean=1.95, range=1.4-2.6). Strong classification agreement between signatures was observed, especially for predicted low-risk patients by adenocarcinoma-derived signatures. Expression of proliferation-related genes correlated strongly with GEP subtype classifications and RP scores, driving the gene signature association with prognosis. A three-group consensus definition of samples across 10 GEP classifiers demonstrated aggregation of samples with specific smoking patterns, gender, and *EGFR*/*KRAS* mutations, while survival differences were only significant when patients were divided into low- or high-risk. In summary, our study demonstrates a consensus between GEPs and RPs in lung adenocarcinoma through a common underlying transcriptional program. This consensus generalizes reported problems with current signatures in a clinical context, stressing development of new adenocarcinoma-specific single sample predictors for clinical use.

## INTRODUCTION

Lung cancer is the leading cause of cancer death worldwide, with adenocarcinoma as the largest histological subtype [1, 2]. During the last decade, significant advances have been made in understanding the molecular characteristics of lung adenocarcinoma. Some of these discoveries have been translated into new therapeutic options, with targeted treatments for patients with tumors harboring *EGFR* mutations or *ALK* gene fusions representing success stories. Unfortunately, while the new gene targeted treatments initially show an often-dramatic patient benefit, patients eventually relapse. The tumor-node-metastasis (TNM) staging system currently represents the best prognostic factor for non-small cell lung cancer (NSCLC) patients in clinical use. However, even for NSCLC patients with the best prognosis, resectable stage I disease, approximately 30% will relapse with a 5-year survival rate of 58-73% [3]. This heterogeneity in the clinical course of patients with the same tumor stage stresses the need for additional biomarkers that can improve prognostication and prediction of response to therapy in lung adenocarcinoma.

Gene expression profiling has been used extensively to divide early stage lung adenocarcinoma into different gene expression phenotypes (GEPs), each comprising of two or more subtypes, associated with clinicopathological characteristics such as smoking, tumor stage and mutational patterns, and to derive prognostic and/or predictive risk predictors (RPs) (see e.g. [4-14] and [15, 16] for review). Such gene signatures hold promise for clinically useful and molecularly driven disease stratification independent of current prognostic variables, provided adequate validation. However, in lung adenocarcinoma considerable challenges remain before such gene signatures are ready for clinical use (see e.g. [17] for review). First, only a few of the reported prognostic/predictive signatures are available as single sample predictors (SSPs) that can be used for independent validations. Notably, only a few studies reporting prognostic/predictive signatures have attempted to reproduce previously reported signatures for comparison, often with hardship and focused only on prognostic performance as evaluation criteria (see e.g. [9, 17]). Consequently, most GEP and RP signatures in lung adenocarcinoma have not been carefully investigated for classification robustness, prognostic value, and treatment predictive value in large multicohort analyses. For one lung cancer GEP [5] and one RP [9] signature it has recently been shown in multicohort analyses that the common usage of a gene-centering / data normalization step in the classification procedures can effect robustness in subtype assignment or risk score calculation, respectively [18, 19]. Second, the agreement between different adenocarcinoma gene signatures in terms of subtype and risk prediction classification has not been systematically investigated. It has been hypothesized that across different GEPs there is a main division of tumors into a terminal respiratory unit (TRU) like subtype with better patient outcome and a second non-TRU subgroup with a poorer patient outcome [20]. The TRU subgroup is enriched for female gender, never-smokers, and tumors that express the TITF-1 transcription factor and surfactant proteins, and show morphological similarity to type II pneumocytes, Clara cells, and nonciliated bronchioles [20].

To address the questions of classification consensus, common underlying transcriptional programs, and associations with patient outcome for different GEPs and RPs in lung adenocarcinoma, we conducted a pan-signature consensus analysis of 18 reported GEPs and risk stratification signatures in 17 cohorts comprising 2395 transcriptional profiles. We show that the majority of signatures represent a relevant prognostic patient stratification in lung adenocarcinoma, and that significant classification agreement exists between signatures, especially in the group of patients for whom better outcome is predicted. Expression of proliferation-related genes was identified as the main biological process associated with the agreement between low- and high-risk groups predicted by the majority of gene signatures. Together, our results provide a general insight into the nature and agreement of GEP and RP signatures in primary lung adenocarcinoma, important for the understanding of their prognostic value.

## RESULTS

### GEP and RP classification of lung adenocarcinoma

The clinical characteristics of the 2395 adenocarcinoma patients are shown in Tables 1, and S1 and have been reported previously [18]. We classified the 2395 tumors according to 18 lung adenocarcinoma GEP and RP signatures, and one *in silico* derived 155-gene breast cancer proliferation signature (Table 2 and Supplementary Methods for explicit details). The latter signature was used to correlate classification results from lung cancer derived signatures to a classification based on proliferation-related genes displaying highly correlated expression unrelated to lung tissue. For several GEP signatures, only lists of significant/predictive genes discriminating subtypes were readily available from original studies and not specific predictive models including weights and cut-points (e.g. Park et al. [21]) (see Supplementary Methods for detailed information about each signature). For these signatures we used a classification approach including consensus clustering or k-means clustering to assign samples to signature subtypes. We acknowledge that our implementation of some described signatures may thus not be identical for every individual sample as in the original studies. Despite these potential differences in classification of individual samples, the approach taken provides a way of systematically comparing signatures for analysis of broader patterns and associations.

**Table:** Clinical characteristics of the total cohort of patients with adenocarcinoma

	Total cohort*
**Number of patients**	2395
**Number of cohorts**	17
**Stage**	
I	63%
II	20%
III	15%
IV	2%
**Sex**	
Female	53%
Male	47%
**Age, median (range)**	64 (21-91)
**Smoking status**	
Never-smoker	24%
Smoker	76%
**Mutation status**	
*EGFR*	29%
*KRAS*	23%
Number of patients with adjuvant chemotherapy** (%)	176 (7.3%)
**Outcome**	
Overall survival (OS), median (years)	2.94
OS number of events (% of all patients)	38%
Distant metastasis-free survival (DMFS), median (years)	3
DMFS number events (% of all cases with DMFS data)	42%

* Characteristics are presented as percentages of all cases with available annotations.

** According to original studies, including only patients with available outcome data.

**Figure 1:**
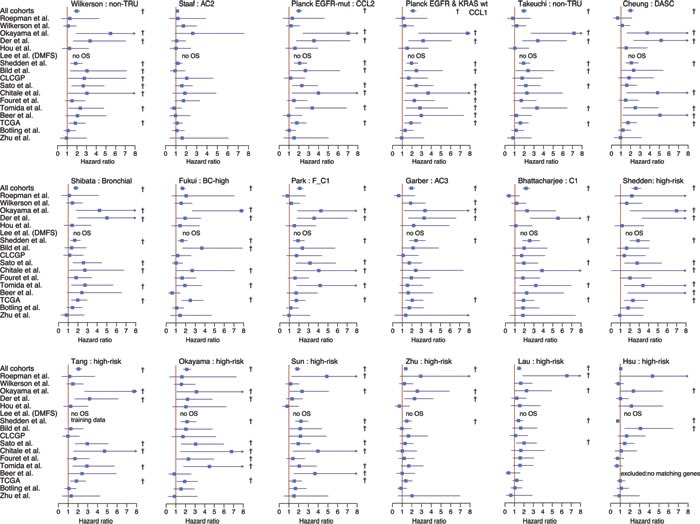
Association of gene expression signature classifications with patient outcome in lung adenocarcinoma 2395 tumors across 17 cohorts were classified according to 18 different gene expression phenotypes or risk predictors. Association of classifications with patient outcome was investigated using univariate analysis with overall survival as the endpoint and included patients of all stages of disease. Bars for each cohort and subtype represent hazard ratios with 95% confidence intervals. For Wilkerson et al. [5], phenotype analysis was restricted to TRU *versus* non-TRU classified tumors (merged group of Proximal-proliferative and Proximal-inflammatory). For other multiclass signatures, such as Garber et al. [8], hazard ratios are shown for the subtype with the most prominent poor outcome association. With the exception of the Staaf et al. AC2 subgroup and the Bhattacharjee et al. C1 subgroup, all subtypes displayed represent high-risk groups from the original studies. For all subtypes, the reference group is the original low-risk group (see Table 2). † indicates a significant *p*-value (*p* < 0.05) in the univariate analysis. TRU: terminal respiratory unit, DASC: distal airway stem cell.

To investigate the accuracy of our classification approach compared to the original studies, we performed univariate survival analysis (including patients of all stages) using patient overall survival as endpoint for each signature in both the total and each individual cohort. We observed that 16 out of 18 GEPs and RPs were significantly associated with patient overall survival in the entire cohort (*p* < 0.05), as well as in several individual cohorts, in a way consistent with claims in the original studies (Figure 1). Hazard ratios (HR) for the significant signatures in the total cohort ranged between 1.4-2.6, with average HR = 1.95±0.3 (standard deviation). For one of the signatures not associated with outcome in the total cohort, the AC1/AC2 signature previously reported by our group [12], the insignificant result is consistent with this signature being reported to be associated with smoking status, a variable related to outcome in only one of the included public cohorts with available data (see ref [18]). While association with patient outcome was observed in several individual cohorts for different signatures, no GEP or RP signature was associated with patient outcome in the Botling et al. [22], Hou et al. [23], or Zhu et al. [9] cohorts. In addition, in the CLCGP [24] and Fouret et al. [25] cohorts only one and two signatures, respectively, showed univariate significance (*p* < 0.05) (Figure 1).

**Table:** Investigated adenocarcinoma gene expression phenotype signatures / risk prediction models

Gene expression signature	Ref	Origin ^A^	Signature type ^B^	Number genes/probes	Signature subtypes
Wilkerson et al.	[5]	AC	GEP: Centroid classifier	506	TRU, Proximal-proliferative*, & Proximal-inflammatory*
Staaf et al.	[12]	AC	GEP: Centroid classifier	176	AC1 & AC2
Planck et al.	[13]	AC	GEP: Centroid classifier	746	EGFR mut low-risk & EGFR mut high-risk*
Planck et al.	[13]	AC	GEP: Centroid classifier	871	EGFR & KRAS wt low-risk & EGFR & KRAS wt high-risk*
Takeuchi et al.	[4]	AC	GEP: Gene signature	293	TRU & non-TRU-like*
Cheung et al.	[37]	AC	GEP: Gene signature	249	Alveolar & distal airway stem cell (DASC)-like*
Shibata et al.	[38]	AC	GEP: Gene signature	78	Alveolar & bronchiolar*
Fukui et al.	[36]	AC	GEP: Gene signature	1829	BC-low & BC-high*
Park et al.	[21]	AC	GEP: Gene signature	193	S_C1 & F_C2*
Garber et al.	[8]	AC	GEP: Gene signature	146	AC1, AC2, & AC3*
Bhattacharjee et al.	[31]	AC	GEP: Gene signature	100	C1, C2*, C3, & C4
Shedden et al., method A	[11]	AC	Risk predictor	13830	Low-risk, moderate-risk, high-risk*
Tang et al.	[28]	AC	Risk predictor	18	Low-risk, high-risk*
Okayama et al.	[29]	AC	Risk predictor	4	Low-risk, moderate-risk, high-risk*
Sun et al.	[39]	AC	Risk predictor	50	Low-risk, high-risk*
Zhu et al.	[9]	NSCLC	Risk predictor	15	Low-risk, high-risk*
Lau et al.	[26]	NSCLC	Risk predictor	3	Low-risk, high-risk*
Hsu et al.	[30]	NSCLC	Risk predictor	4	Low-risk, high-risk*

A Origin of the signature. AC: adenocarcinoma, NSCLC: NSCLC.

B Explicit details on classification procedures are described in Supplementary Methods. GEP: Gene expression phenotype signature.

* Denoted as high-risk / poor outcome group(s) in original studies.

**Figure 2:**
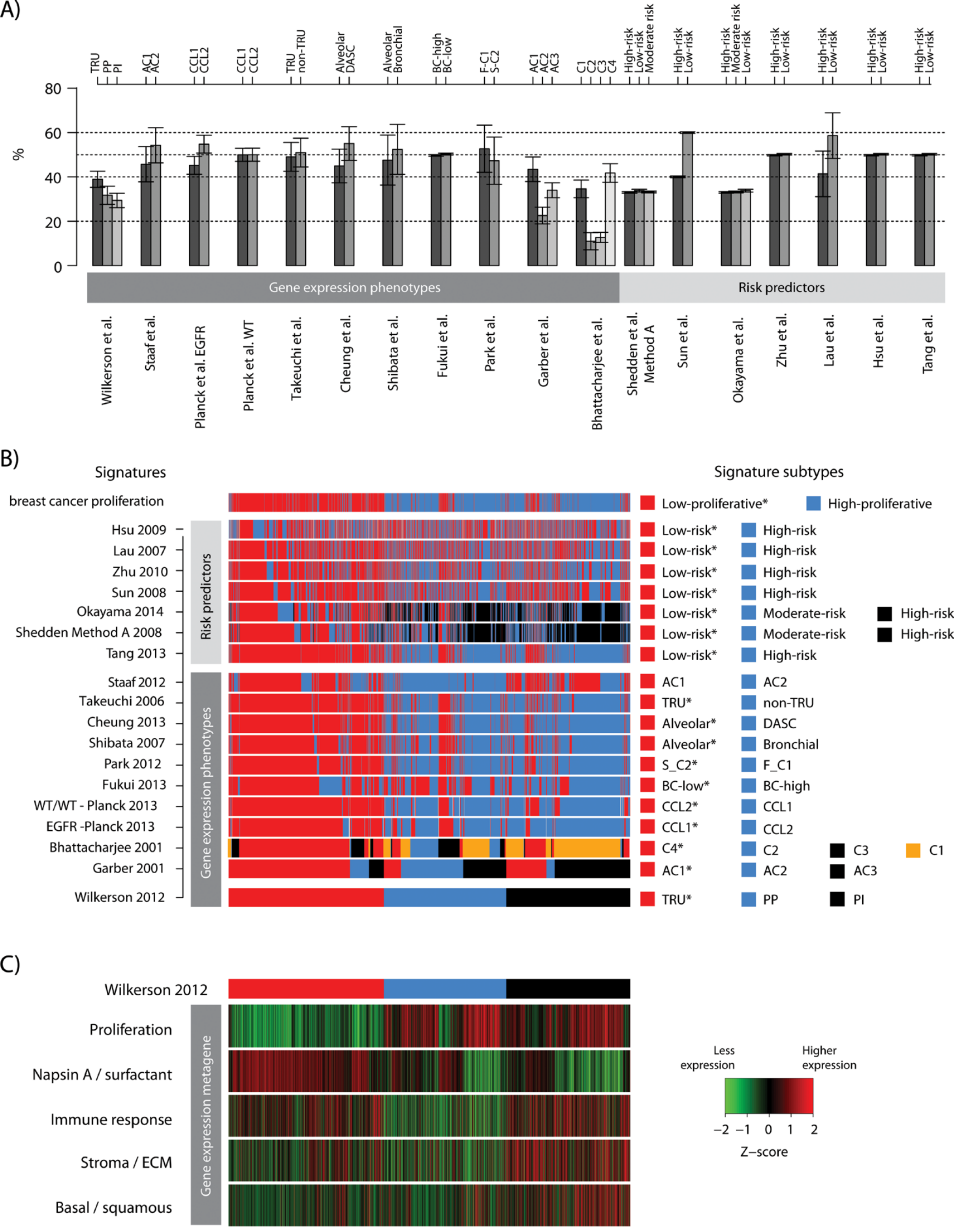
Consensus of gene expression phenotype and risk predictor classification of lung adenocarcinoma 2395 tumors across 17 cohorts were classified according to 18 different lung cancer gene expression phenotypes (GEPs) and risk predictor (RPs) models and one breast cancer proliferation signature. **A.** Proportions of each lung cancer GEP and RP subtype are shown across the 17 cohorts. Bars represent the mean of subtype proportions for each signature with standard deviations across all cohorts. A low standard deviation implies that similar subtype proportions were observed across cohorts. Subtype names, as in the original reference, are shown along the top of the panel. **B.** Consensus of GEP and RP classifications for the total cohort. Signature subtypes (right legend) were overlaid for each signature (row) across samples (columns), and ordered according to the Wilkerson et al. [5] signature as an illustration of classifications performed. Classification according to a breast cancer proliferation signature is also shown to demonstrate agreement of classification with a division based on expression of proliferation-related genes. A * indicates a good outcome group as reported in the original reference. **C.** Corresponding expression of metagene scores from five gene expression metagenes representing different biological processes [35] in the total cohort. Sample ordering (columns) corresponds to panel B, with the top bar representing the Wilkerson et al. classification for comparison with panel B. Metagene scores are shown as Z-transformed values. Transformation was performed within each cohort prior to combining all samples. ECM: extra cellular matrix.

All investigated signatures showed a strong similarity in the proportions of signature subtypes across individual cohorts (Figure 2A and Supplemental Figure S1 for more explicit details). For all RPs, except Lau et al. [26], the similar proportions are expected, as the classification set-up by default divides cohorts into equally sized groups. For the GEP signatures that divided tumors into two groups based on centroid prediction or consensus clustering, subtype proportions were generally quite balanced (50/50% split). For GEP signatures with >2 subtypes (the Wilkerson, Garber and Bhattacharjee signatures), proportional differences between subtypes were larger, i.e., not apparently balanced (e.g., a 33/33/33% split) (Figure 2A). However, across cohorts the standard deviations in individual subtype proportions for all GEP signatures were fairly low, meaning that equal proportions of samples were assigned to the individual subtypes of a signature, irrespective of cohort size or composition (Figure 2A and Supplementary Figure S1 for details). Notably, these observations are consistent with findings from our recent study, based on the same patient cohort, investigating classification robustness of the Wilkerson et al. GEP signature [18]. Importantly, these results support others and our observations that current SSPs tend to predict subtypes in similar proportions across cohorts [18, 19, 27]. Together, these findings show that the majority of signatures, based on original classification schemes, represent a relevant prognostic division of adenocarcinoma in a general context.

### Gene signature consensus in lung adenocarcinoma

**Figure 3:**
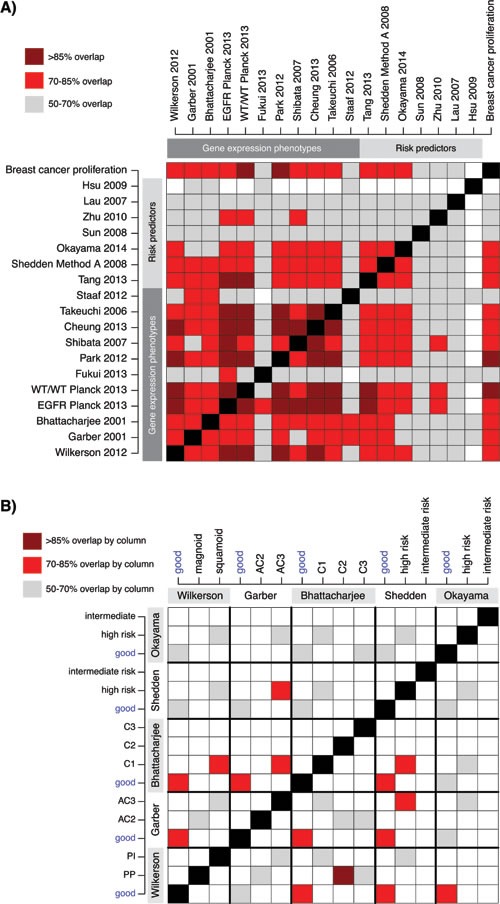
Classification agreement between GEP and RP signatures across 2395 adenocarcinomas **A.** Classification overlap analysis for 18 GEP and RP signatures transformed into two classes of putative “good” and “poor” outcome. For original two-class signatures the annotations in Figure 2B was used for the 2395 patients. For three- or four-class signatures the “good” class was the class originally reported as having best patient outcome (see Figure 2B), while the “poor” class comprised of the remaining classes. Colored cells indicate the agreement in classification (%) between pairs of signatures based on a 2x2 contingency table. **B.** Agreement in classification between three- and four class signatures specifically. Each colored cell displays a percentage of agreement between signature pairs. Frequencies are calculated column-wise, i.e. the sum of patients with a column class is used as denominator for each signature pair comparison. E.g. nearly all (>85%) C2 classified cases by Bhattacharjee et al. [31] (column), are classified as proximal-proliferative, (PP), by Wilkerson et al. [5] (row). Reported good outcome / low-risk groups are highlighted.

To investigate whether agreement exists between the classifications of the samples obtained with the 18 signatures, we first overlaid the classifications from all signatures together with a breast cancer derived proliferation classifier (Figure 2B). We observed strong agreement between the classification of the different good outcome / low-risk groups, especially for the GEP signatures and the Tang et al. [28] RP signature, as illustrated in Figure 2B by the consistent classification agreement of each signature with the Wilkerson et al. [5] TRU subtype (a proposed low-risk group) signature selected as a visualization baseline. Consequently, poor outcome / high-risk or moderate-risk patients were predominantly classified as non-TRU (i.e., Proximal-proliferative or Proximal-inflammatory) by the Wilkerson et al. centroid classifier. Interestingly, the consensus in prediction of a low-risk group between GEPs and RPs that were derived specifically from adenocarcinoma samples (refs [11, 28, 29], Table 2) appeared to be better than between GEPs and RPs derived from NSCLC cohorts (like the Lau and Hsu et al. [26, 30] RP signatures). Moreover, while there is strong agreement in classification between the two-group GEP signatures for all outcome subgroups, there seems to be less agreement for subgroups with poorer outcome between GEP signatures with >2 subtypes (such as the Wilkerson et al. [5], Garber et al. [8] and Bhattacharjee et al. [31] signatures). To further substantiate these observations we performed an extensive analysis of signature pair classification overlap (Figure 3). First, we transformed all signatures into two-class signatures of good and poor outcome using reported class annotations from original studies and calculated the overlap between each signature pair (see Figure 3A for details regarding the transformation of three- and four-class signatures). Notably, for the majority of signatures derived in adenocarcinoma (GEPs and RPs) we observed an agreement above 70%, and even >85% for a set of signatures, while classification overlap was poorer for RP signatures derived in NSCLC cohorts (Figure 3A). Next, we analyzed the overlap for three- and four-class signatures specifically. Here the mapping between different classes is more difficult. For this analysis we renamed the classes with reported best outcome as “good” similar to the previous analysis and compared classification overlap between all class combinations (Figure 3B). Notably, the class combinations with the best classification overlap between different signatures were often the good outcome groups, often showing >70% overlap. For remaining classes, less agreement compared to the two-class case was observed (compare Figure 3A and 3B).

Next, we compared classification results of the different lung cancer signatures with the breast cancer derived proliferation signature, with the aim to investigate the agreement between lung cancer subtype and risk prediction groups with a classification based solely on expression of proliferation-related genes derived from another tumor disease. Notably, a clear agreement of the lung adenocarcinoma classifications with the breast cancer proliferation classification was observed (Figure 2B), suggesting that the main transcriptional driver of classification for the lung signatures is associated with expression of proliferation-related genes. Specifically, low-risk groups seem to be characterized by lower expression of proliferation-related genes than in the poor outcome / high-risk groups. This was further substantiated in the comprehensive signature pair classification overlap analysis, demonstrating a powerful consistency in classification into groups of better or worse prognosis with the expression of proliferation-related genes (the breast cancer proliferation classification) (Figure 3A). Finally, we compared the expression of the breast cancer proliferation signature to that of other reported proliferation signatures, like the CIN70 by Carter et al. [32], and the cell cycle progression (CCP) signature originally derived in prostate cancer [33] and later validated in lung adenocarcinoma [34]. For the 2395 samples we observed Pearson correlations ≥0.97 between the three signature scores, implying that any associations made for the breast signature is also valid for the other signatures.

Taken together, these results suggest that investigated signatures, especially if derived in adenocarcinoma cohorts, generally agree on whether an adenocarcinoma should be classified as low-risk or not, coinciding with its expression of proliferation-related genes.

### Underlying transcriptional processes behind GEP and RP consensus

To compare the analyzed gene signatures we first analyzed the overlap of unique genes between the signatures (Supplementary Figure S2). This comparison demonstrated a low gene overlap between the majority of signature combinations (< 25%), excluding Shedden et al. [11] (the largest signature, *n* = 9591 genes), and a stronger overlap found between the two Planck et al. [13] signatures with the Park et al. [21] and Tang et al. [28] signatures (50-75%), and between Park et al. and Wilkerson et al. To substantiate our hypothesis that proliferation is a key component in the consensus between the GEP and RP signatures, and is a likely explanation of their association with prognosis, we analyzed the expression of five lung cancer derived transcriptional metagenes representing different biological processes [35], *versus* the signature classifications in the 2395 samples. This analysis demonstrated that tumors classified as low-risk across the GEP and RP signatures typically show less expression of proliferation-related genes (Figure 2C compared with 2B). However, as we recently demonstrated in our analysis of the Wilkerson et al. GEP signature [18], expression of proliferation-related genes, approximated by the proliferation metagene, resembles a unimodal distribution of continuous values in adenocarcinoma making associations with GEP and RP subtypes dependent on arbitrary cut-offs.

**Figure 4:**
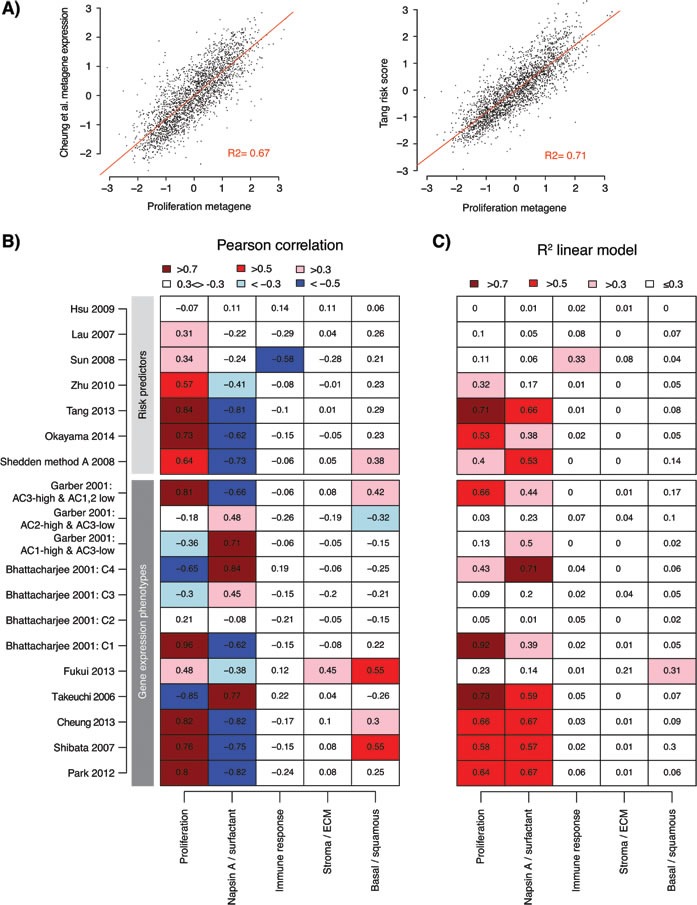
Association of gene expression phenotypes and risk predictor scores with transcriptional metagenes representing different biological processes **A.** Example of correlation between expression of a GEP signature metagene (Cheung et al. [37]) *versus* the expression of the proliferation metagene from Karlsson et al. [35] (left), and correlation between risk scores from a lung adenocarcinoma derived RP signature (Tang et al. [28]) and the proliferation metagene (right) across the 2395 adenocarcinomas (points). Red line shows the fit of a linear regression model, together with the calculated R^2^ value. Displayed data values represent Z-transformed expression values or risk scores to account for variability across cohorts. **B.** Pearson correlation of gene expression signatures with expression of five lung cancer transcriptional metagenes representing different biological processes [35] across 2395 adenocarcinomas in 17 cohorts. For each cohort and signature / process, a metagene expression value was calculated for the signature or process as described in Supplementary Methods and transformed to a Z-score. For risk predictors, risk scores were Z-transformed. Pearson correlation was calculated for the total cohort for each gene signature / predictor *versus* the biological processes. Some gene signatures are comprised of multiple subset signatures from the original studies. **C.** The fit (represented by the the R2 value) of a linear regression of Z-score values from gene signatures *versus* the biological processes, as exemplified in panel A, shows the strength of the linear relationship between signatures (or subcomponents of signatures) and expression of metagenes. ECM: extra cellular matrix.

We next asked how specific gene signatures (or reported subcomponents of gene signatures) from all gene signature based GEPs (*n* = 7, excluding centroid-based signatures) and all RPs (*n* = 7) correlated with the five metagenes [4, 8, 9, 11, 21, 26, 28-31, 36-39] (Figure 4A and 4B). The strongest positive correlations for the analyzed gene signatures were observed for the proliferation metagene. For RP signatures this positive correlation means that an increasing risk score correlate with an increasing proliferation. The strongest negative correlations were observed for the Napsin A / surfactant metagene, which by itself is anti-correlated with the proliferation metagene (see ref [35]). Moreover, all analyzed GEP signatures seemed to carry a proliferation-related component, and all RPs, except Hsu et al. [30], showed correlation between risk scores and the proliferation metagene (Figure 4B). While a few additional strong correlations with other metagenes were observed, such as the correlation between the Shibata et al. [38] signature and the basal / squamous metagene, the fit of a linear regression model (represented by the R^2^ value) for these correlations was notably poorer than for the proliferation and Napsin A / surfactant metagene correlations (Figure 4C and Supplementary Figure S3). This indicates that the associations for these relationships are less linear than for the proliferation metagene, and that the observed agreement between different GEPs and RPs can be explained by, at least one, common transcriptional program, i.e., expression of proliferation-related genes.

### Molecular and clinicopathological characteristics of GEP consensus groups

**Figure 5:**
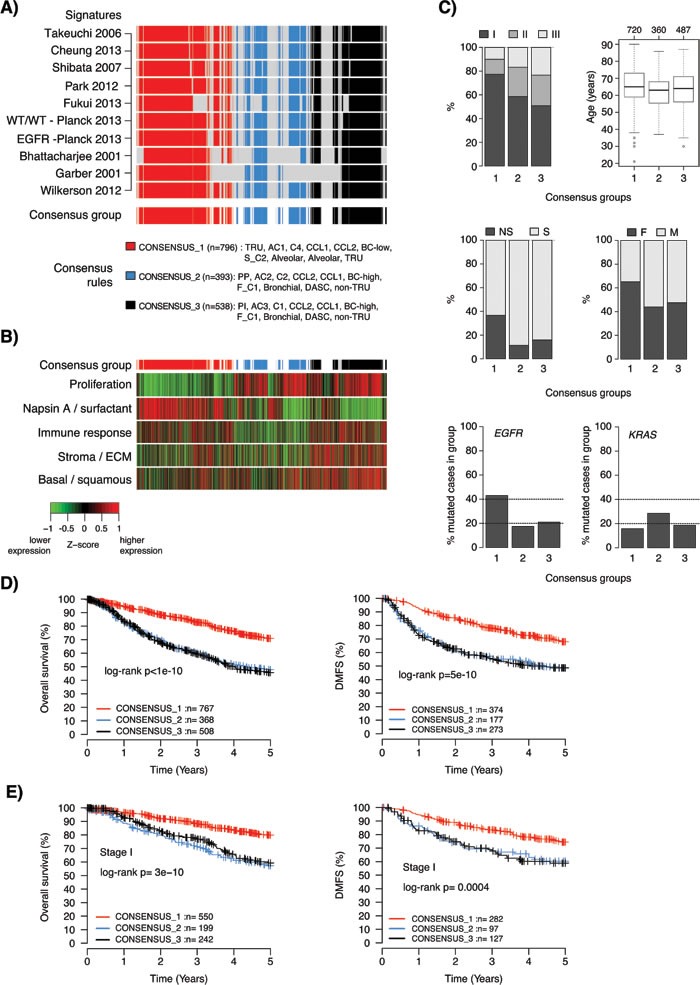
Characteristics of consensus samples across lung adenocarcinoma gene expression phenotypes **A.** Definition of three consensus groups across 10 reported lung adenocarcinoma GEP signatures. Rows represent individual signatures and columns represent samples. Consensus groups (bottom row) were defined as specified in the figure, and required a consensus classification in ≥8 of the 10 signatures. In total, 72% (*n* = 1727) of samples in the total cohort were assigned to a consensus group. **B.** Corresponding expression of metagene scores from five expression metagenes representing different biological processes [35]. Sample ordering (columns) corresponds to that shown in panel A. Metagene scores are shown as Z-transformed values. Z-score transformation was performed within each cohort prior to combining all samples. The top bar indicates the consensus groups according to that shown in panel A. **C.** Distribution of tumor stage (data for *n* = 1545 cases), age (*n* = 1567 cases), smoking status (*n* = 1374 cases), gender (*n* = 1646 cases), *EGFR* mutations (*n* = 822 cases with mutation status yes/no), and *KRAS* mutations (*n* = 885 cases with mutation status yes/no) for the three consensus groups. **D.** Kaplan-Meier survival plots of consensus groups for all consensus cases using overall survival (left) or distant metastasis-free survival (DMFS) (right) as endpoints. **E.** Kaplan-Meier survival plots of consensus groups for consensus cases with stage I disease using overall survival (left) or DMFS (right) as endpoints. TRU: terminal respiratory unit, PP: Proximal-proliferative, PI: Proximal-inflammatory, DASC: distal airway stem cell, NS: never-smoker, S: smoker, F: female, M: male.

To investigate if our consensus analysis of different GEP classifiers could refine existing transcriptional adenocarcinoma subgroups regarding important clinicopathological and mutational variables, we defined consensus groups comprising of samples with high classification agreement across multiple GEP signatures. Due to the focus on patient outcome for these new/refined groups we excluded the AC1/AC2 classifier by Staaf et al. [12], as this signature was not associated with overall survival in the total cohort. Based on rules of subtype classification relationships for the remaining 10 GEP classifiers and the assumption of three groups (CONSENSUS_1, CONSENSUS_2, CONSENSUS_3), we identified samples (*n* = 1727 in total, 72%) with consensus for ≥8 classifiers (Figure 5A and Supplementary Methods). Division into three consensus groups implies additional stratification of analyzed two-class signatures. This stratification divided the high-risk groups in these signatures into two subgroups (see legend Figure 5A). A high degree of consistency was observed in the fraction of cases assigned to each consensus group within an individual cohort, with an overall median fraction across all cohorts of 47.5% for CONSENSUS_1 (standard deviation (SD) = 4%, interquartile range (IQR) = 4.8%), 22.5% for CONSENSUS_2 (SD = 5.7%, IQR = 6.4%), and 30.5% for CONSENSUS_3 (SD = 5.9%, IQR = 7.2%).

The consensus groups were characterized by notable differences in expression of the five metagenes representing different biological processes [35], consistent with the previous analyses (Figure 5B). Specifically, the CONSENSUS_1 group was characterized by lower expression of proliferation-related genes and higher expression of Napsin A / surfactant genes, while the opposite pattern occurred in the CONSENSUS_2 and CONSENSUS_3 groups. Moreover, CONSENSUS_3 was characterized by higher expression of stromal / ECM, immune response associated genes and basal / squamous like genes. Finally, CONSENSUS_2 had generally the lowest expression of immune response associated genes and stromal / ECM associated genes. These findings suggest that CONSENSUS_2 tumors may harbor less, and CONSENSUS_3 more, infiltrating non-malignant cells, as the expression of these immune response associated and stromal / ECM metagenes typically correlate with infiltration of lymphocytic and stromal cells. To substantiate this hypothesis we analyzed pathological tumor purity estimates for 158 included cases from The Cancer Genome Atlas (TCGA) study [14]. Accordingly, CONSENSUS_3 samples showed lower tumor purity estimates than the other groups (Kruskal-Wallis test *p* = 0.001).

Next, we examined the consensus groups for trends in clinicopathological characteristics. The CONSENSUS_1 group showed an enrichment of lower stage tumors (Chi-square test *p* = 1x10^-20^), never-smokers (*p* = 9x10^-22^), female patients (*p* = 3x10^-14^), and *EGFR* mutations (*p* = 2x10^-12^) (Figure 5C). While the enrichment of *EGFR* mutations in a subgroup comprising of many never-smokers is in line with the literature, the observed frequency of ~40% *EGFR* mutations in CONSENSUS_1 is partly driven (skewed) by a high mutation rate in two included cohorts, Okayama et al. [40] and Fouret et al. [25] (56% and 49% mutation rate, respectively). However, for the 158 cases included in the TCGA study [14] nearly 75% of CONSENSUS_1 cases were referred to as oncogene-positive in the TCGA study, meaning that they harbored a known activating RTK/RAS/RAF pathway somatic event. These associations suggest that the CONSENSUS_1 tumors could be more dependent on oncogene activation than tumors in the other groups. Together, the characteristics of the CONSENSUS_1 group are well in line with a proposed TRU-like subtype of adenocarcinoma reported previously (Chi-square test *p* = 0.005) [4, 5, 14].

The CONSENSUS_2 group is characterized by slightly younger patients (Kruskal-Wallis test *p* = 2x10^-5^) and a higher frequency of *KRAS* mutations (Chi-square test *p* = 0.0007) (Figure 5C). Summarized mutational data obtained from the TCGA study [14] and Karlsson et al. [41] revealed that CONSENSUS_2 and 3 cases showed significantly more mutations overall (Kruskal-Wallis test *p* = 2x10^-11^), more nonsilent mutations per mb sequence (Kruskal-Wallis test *p* = 5x10^-5^), and a higher frequency of C>A mutation transversions (a smoking-related mutation signature [42], Kruskal-Wallis test *p*= 8x10^-5^) than CONSENSUS_1 cases. Together, these mutational characteristics are consistent with a higher fraction of smokers in the CONSENSUS_2 and 3 groups [14, 42], again consistent with previous reports about the characteristics of a non-TRU like adenocarcinoma subtype [4, 5, 14].

Regarding patient outcome, the main difference was between CONSENSUS_1 *versus* 2 and 3, with the former representing a low-risk group and the latter two representing high-risk groups in the total cohort when using either overall survival or distant metastasis-free survival as endpoints (Figure 5D). The same results were observed also for patients with stage I disease specifically (Figure 5E). No significant difference in overall survival between CONSENSUS_2 and CONSENSUS_3 cases was observed in the total cohort (log-rank *p* = 0.74), or in stage I disease specifically (*p* = 0.43). This finding is consistent with similar findings of no difference in patient survival between proximal-proliferative and proximal inflammatory subtypes based on the Wilkerson classifier (see [18]).

In summary, these analyses suggest that while multiple subgroups defined by consensus-classified cases across multiple GEP signatures to some extent share clinicopathological traits and prognosis, a relevant prognostic division can be made already between a TRU-like and a non-TRU like subgroup.

## DISCUSSION

Improved molecular characterization and stratification of lung adenocarcinoma is important for prognostication and identification of new predictors of treatment response in order to improve patient outcome. Although gene expression profiling studies since more than a decade have identified numerous molecular phenotypes and prognostic and/or chemotherapy predictive signatures in adenocarcinoma (see e.g. [15, 16] for a review), a thorough consensus analysis of a large number of reported GEP and RP signatures has not been performed. We performed a comprehensive multicohort pan-signature analysis of 18 GEP and RP signatures in 2395 patients, showing that: i) the majority of signatures represent a relevant prognostic division of lung adenocarcinoma based on original classification schemes, ii) signatures generally display a low gene overlap in line with previous reports (see, e.g., [9, 26, 43]), iii) there is overall good agreement between many signatures in defining a low-risk group of patients with molecular and clinicopathological characteristics similar to those reported for TRU-like adenocarcinoma, iv) signatures derived in adenocarcinoma generally display better classification agreement than signatures derived in mixed NSCLC cohorts, v) expression of proliferation-related genes is one of the key components behind prognostic associations and classification agreement in almost all GEP and RP signatures (especially for adenocarcinoma derived signatures), and vi) a low-risk TRU and a high-risk non-TRU division of adenocarcinoma seems to be the most prognostically relevant division by molecular phenotype based on consensus agreement between signatures. Taken together, our results provide increased understanding of GEP and RP gene signatures performance, consensus, and prognostic values in lung adenocarcinoma.

Comparison of classifications from the 18 GEPs and RPs demonstrated significant agreement between many signatures, especially in assignment of cases to low-risk subgroups (Figures 2B and 3). Cases classified as low-risk by different GEP and RP signatures showed lower expression of proliferation-related genes, and coincided well with a 50/50% split of cases by a breast cancer derived proliferation score, or as we also demonstrated with proliferation signatures derived in other diseases [32, 33]. Moreover, nearly all investigated signatures/classifiers included a proliferation component or correlated strongly with the expression of proliferation-related genes (Figure 4), despite a generally low gene signature overlap (Supplementary Figure S2). The latter observation is in perfect agreement with previous reports in lung cancer [9, 26, 43] and analysis of published and random prognostic gene signatures in breast cancer [44]. Together, these results pinpoint that proliferation is the main driving force behind this major split of subtype classifications in lung adenocarcinoma, affecting also patient outcome. Provocatively, this conclusion also suggests that random gene signatures with high prognostic value can be defined in lung adenocarcinoma, as long as the signature genes correlate with expression of a few proliferation-related genes, similar to what has been described in breast cancer [44]. Our identification of a low-risk consensus group across 10 different GEP signatures further show that low-risk adenocarcinomas typically have higher expression of surfactant proteins and Napsin A, and are associated with clinicopathological characteristics such as lower tumor stage, never-smoking, female gender and oncogene activation (including *EGFR* mutations) (Figure 5). It should be noted that while our identification of consensus groups across many different signatures may render general patient groups that are potentially a bit more robust compared to individual classifiers, this analysis is still affected by the problems of data centering noted by us and others [18, 19, 27] (as it is a mere summarization of individual classifications). Overall, these results are in excellent agreement with the literature, and the hypothesis by Yatabe et al. [20] of a main division of adenocarcinoma into a TRU-like subtype and a non-TRU subgroup with a worse patient outcome, a terminology also used for the subtypes defined by the Wilkerson et al. [14] and Takeuchi et al. [4] GEP signatures. However, whether reported GEP and RP signatures also represent significant predictors of prognosis or treatment response in patients with non-operable disease (accounting for 66-75% of all lung cancer cases) remains to be determined, partly because of the scarcity of gene expression studies based on advanced stage lung adenocarcinoma.

Less agreement was observed for non-TRU samples classified to different high-risk subtypes by GEP [5, 8, 31] and RP [11, 29] classifiers with >2 subtypes/classes (Figures 2B, 3 and 5A). For the GEP signatures, the poorer agreement may be due to that other (potentially signature specific) expression programs, besides a generally higher expression of proliferation-related genes, define non-TRU subtypes in these signatures. For the two RP signatures, subgroups are formed based on arbitrary cut-offs in a continuous variable, i.e. the risk score, or by association; expression of proliferation-related genes. Consequently, selection of different (and more optimized) quantile cut-offs could easily improve overlap with different GEP signatures, and potentially also improve the prognostic association. Interestingly, while our consensus analysis defined groups of adenocarcinomas with high classification agreement across high-risk GEP subtypes, these consensus groups did not differ in outcome, consistent with our recent findings for the Wilkerson et al. non-TRU subtypes specifically [18]. While not specifically addressed in this study, the question of the number and nature of reproducible gene expression phenotypes in lung adenocarcinoma clearly remains to be resolved.

The GEP signatures in the current study have been derived through different types of unsupervised analyses (typically hierarchical clustering of genes with large differences in expression across analyzed tumors). Ideally, this type of analysis defines molecular subgroups based on distinct transcriptional programs, thereby making the molecular subgroups powerful explainers of the observed transcriptional variation across sets of tumors. Unsupervised clustering of mRNA expression levels in NSCLC tumors represents a powerful example, resolving the histological subgroups as different transcriptional clusters (see e.g. [8, 22, 24, 31]). Acknowledging the type of analyses performed in the original GEP studies has two implications for the broader interpretation of our findings. First, it may provide insight into the somewhat poorer agreement and prognostic performance we observed for RP signatures originally developed in NSCLC cohorts [9, 26, 30] compared to signatures derived strictly in adenocarcinoma. Specifically, NSCLC derived RP signatures may not capture the dominating prognostic transcriptional component in adenocarcinoma (proliferation) equally well as signatures derived strictly in adenocarcinoma. This is because training and feature selection (genes whose expression are associated with patient outcome by, e.g., regression analysis) is performed in cohorts with mixed transcriptional characteristics (different tumor histologies) driving the feature selection towards different gene sets. This hypothesis is supported by: i) several adenocarcinoma derived RPs that have a strong proliferative component/association are not prognostic in squamous cell carcinoma (see e.g. [28, 29, 45]), ii) reports of overall higher expression of proliferation-related genes in squamous cell carcinoma than adenocarcinoma [13], and iii) weaker associations of risk scores from NSCLC derived signatures with expression of proliferation-related genes (Figure 4). Consequently, prognostic gene signatures may be more successful if derived and applied separately within histological subtypes. Second, while our identified consensus groups across different GEP signatures were associated with key clinicopathological and molecular variables in adenocarcinoma, such as smoking status, tumor stage, gender, and mutational patterns of *EGFR* and *KRAS*, we observed heterogeneity for all these traits across consensus groups (Figure 5). This observed heterogeneity suggests that these variables do not represent/define strong independent gene expression phenotypes, and therefore only explain part of the general transcriptional heterogeneity in adenocarcinoma (as they would otherwise have been detected as their own molecular subtypes). Importantly, this hypothesis is consistent with findings in gene expression studies performed on specific clinicopathological (smoking) or mutation subgroups (*EGFR*/*KRAS*) in lung adenocarcinoma [41, 46, 47].

We observed association with patient outcome for 16 out of the 18 signatures studied in the total cohort based on original signature classification schemes. While the approach used in the present study provides an independent implementation and multicohort validation of several GEP and RP signatures in a large number of retrospective cohorts, the design does not enable a decision on which is the best prognostic signature, as exact implementation and reproducibility of some signature classifiers was difficult (as also noted by Subramanian et al. [17]). In addition, some classifiers were derived from and/or trained in certain cohorts also included in this study, which compromised cohort independence. Moreover, we acknowledge that additional signatures could have been investigated. However, we hypothesize that many such signatures, especially those with strong prognostic power in lung adenocarcinoma and including a proliferative component, or at least correlating with expression of proliferation-related genes, would also agree on the definition of a low-risk patient group similar to the signatures in the current study. Across individual cohorts we observed considerable variation regarding prognostic value for all investigated signatures (Figure 1). Several explanations are conceivable, including cohort or patient characteristics, such as small cohort sizes, potential treatment differences, insufficient patient follow-up time, and/or biased sample selection. However, both the variation in prognostic value across cohorts, and the low variability in subtype proportions across studied cohorts (Figure 2A), is strikingly consistent with our recent study on the prognostic and chemotherapy predictive value of the Wilkerson et al. GEP signature specifically [18]. In that study, we demonstrated that the methodological set-up of the classifier itself was biased towards keeping the proportions of the subtypes similar in each cohort, more or less irrespectively of the sample composition [18]. Similar bias in estimation of risk prediction scores for the Zhu et al. [9] RP signature was recently described by Qi et al. [19], stating that this type of signature is unsuitable for direct application to individual samples. Notably, the majority of current RP signatures in lung adenocarcinoma performs risk stratification in a similar manner, as demonstrated in Figure 2A. Moreover, the instability of conventional hierarchical clustering of microarray data to gene centering and variation in linkage and distance metric methods is well known. Considering all these different pieces of information, we believe the present study provides strong evidence for extending the critical findings about classifier robustness from previous single signature studies into a general context of current gene signatures in lung adenocarcinoma, as well as other histological lung cancer groups. Specifically, we believe the greatest issue with current prognostic and predictive gene signatures lies in the inability to predict samples truly independent of each other, which causes risk assessments to be cohort dependent. As demonstrated previously by both us [18] and Qi et al. [19], classification of cohorts with skewed sample compositions already at baseline can infer proportionally similar high-risk classification calls in a cohort with low intrinsic patient risk as in a intrinsically high-risk patient cohort (see Qi et al. for an example [19]). Thus, in a general context it matters less which GEP or RP signature that is being used, as they all more or less identify a common group of low-risk adenocarcinoma patients (especially in the two-class setting). However, on the individual patient level risk predictions appear more variable between signatures (caused by for instance different methodological dependencies/limitations and arbitrary non-optimized cut-offs), and, importantly, may actually be disconnected from the intrinsic patient risk. Importantly, these findings likely also apply to forthcoming SSPs in lung cancer that do not address the main classification problem, i.e., to be able to predict cases truly independent of other tumors. Taken together, these critical limitations question the current signatures clinical usefulness, and calls for development of new signatures and algorithms to circumvent the problem.

While different GEP and RP signatures themselves have been reported to provide independent prognostic information in addition to current clinical variables (mainly tumor stage), it has been shown in both lung cancer and other tumor malignancies that mixed risk predictors may have even stronger prognostic value [11, 48, 49]. As a synopsis of our findings, our results suggest that an optimal RP signature for lung adenocarcinoma should be: i) histology specific, ii) constructed around a proxy estimate of proliferation (a continuous value to accommodate the unimodal distribution) in combination with clinicopathological variables like tumor stage, iii) derived and validated in concordance with a proposed baseline set of guidelines for gene signature development, validation, and reporting (see ref [17]), and iv) independent from other tumors in a cohort in terms of sample predictions (i.e., a true single sample predictor). Notably, our suggestion of histology specific RP signatures appearing more favorable is consistent with the current therapeutic strategy for treatment of lung cancer, which accounts for histology due to different therapeutic responses and treatment regimens between histological subtypes. In addition to the points made above, a clinically useful gene signature needs to be: i) implementable in clinical laboratories according to regulatory body approved protocols such as the Clinical Laboratory Improvement Amendments (CLIA), which may favor focused gene expression methods able to analyze formalin-fixed and paraffin-embedded (FFPE) tissue, and ii) preferably also predictive of chemotherapy response in order to guide adjuvant treatment (see [16] for discussion). The latter point is highly relevant in operable disease, potentially requiring a different approach in RP signature development. For instance, it may be clinically more relevant to optimize RP cut-offs to identify a smaller group of patients with very low risk of recurrence (e.g., a five year 90-95% recurrence-free survival) that could be spared adjuvant chemotherapy, instead of using arbitrary cut-offs that maximizes hazard ratio scores or separation of Kaplan-Meier survival curves. Such signature optimization could also take into account existing clinicopathological variables, for instance tumor size, age, and stage, to further enhance signature performance, similar to the commercial Prosigna assay in breast cancer (see e.g. ref [50]).

In summary, by providing this consensus of current GEPs and RPs in lung adenocarcinoma we have connected molecular phenotypes, risk predictions, patient outcome and underlying transcriptional programs of the different classifier types not previously demonstrated. Our results provide a general insight into the nature and agreement of GEP and RP signatures in the disease, and their prognostic value.

## MATERIALS AND METHODS

### Gene expression cohorts

Published transcriptional profiles from 17 cohorts comprising 2395 lung adenocarcinomas with available patient outcome data were collected from authors' websites or public repositories as described in the original studies and summarized as described by Ringnér et al. [18]. Included studies were performed in both western and Asian countries, were limited to surgically resected patients, and were reported since 2001. Overall patient characteristics are summarized in Table 1, and available in detail for each cohort in Supplementary Table S1. Concerning chemotherapy, four cohorts had adjuvant chemotherapy data (treatment / no treatment) available, including 322 cases from Shedden et al. [11] (chemotherapy type not explicitly specified), 133 cases from Sato et al. [51] (UT Lung SPORE randomized trial, combination of mainly carboplatin plus taxanes, see also Supplementary Methods), 85 cases from Fouret et al. [25] (cisplatin-based chemotherapy), and 28 adenocarcinomas from Zhu et al. [9] (JBR.10 randomized trial, combination of cisplatin/vinorelbine chemotherapy) as outlined by Ringnér et al. [18]. Specific data on treatment cycles, treatment duration, and chemotherapy doses were not available. In total, 562 patients from these cohorts had associated outcome data (overall survival), and 176 of these patients received adjuvant chemotherapy (Table 1).

### Gene expression analyses

Affymetrix and non-Affymetrix gene expression cohorts were normalized on a per cohort basis as described by Ringnér et al. [18]. Tumors were classified according to 18 different GEPs or RPs [4, 5, 8, 9, 11-13, 21, 26, 28-31, 36-39] derived from microarray analysis of lung adenocarcinoma or NSCLC cohorts, using reported original classification schemes or consensus clustering [52] of gene signatures on a per cohort basis if not otherwise specified (Table 2, Supplementary Table S1, and Supplementary Methods). Specifically, consensus clustering was used when a GEP SSP was not readily available from the original studies. The typical case was when a GEP signature comprised of only a list of significant genes defining the GEP subtypes. Consensus clustering was chosen to improve the robustness of the classifications with respect to sampling variability by using repeated subsampling and clustering to provide quantitative evidence of the cluster stability. To independently assess the connection between classifications by the lung cancer derived signatures and classification by expression of proliferation-related genes only, we derived a 155-gene breast cancer proliferation signature as described in Fredlund et al. [53] using the online GOBO tool [54], and classified all tumors as low-proliferative (average expression of the 155-genes < median) or high-proliferative. Tumors were also scored according to five reported expression metagenes in lung cancer representing different biological processes [35]; proliferation, immune response, basal / squamous, stroma / extra cellular matrix (ECM), and expression of Napsin A / surfactants on a per cohort basis. Here, a metagene represents a set of genes associated with a specific biological process, for which the average expression is determined (considering direction of expression for individual genes) and taken as a measurement of process activity. The purpose of the five metagene scoring was to contrast GEP and RP classifications with estimates of different biological processes in lung adenocarcinoma, not to use them as classification signatures. In addition, we calculated similar metagene scores for seven GEP signatures [4, 8, 21, 31, 36-38] that were only available as gene lists from the original studies to study the correlation between these metagene scores and the metagenes representing biological processes. Detailed explanation of the classification, metagene construction, and scoring procedures used are available in the Supplementary Methods.

### Survival analyses

Survival analyses were performed in R using the survival package with overall survival or distant metastasis-free survival as endpoints. Survival curves were compared using Kaplan-Meier estimates and the log-rank test. Hazard ratios were calculated through univariate Cox regression. Due to differences in patient follow-up time between cohorts, a five-year censoring was used in all analyses.

## SUPPLEMENTARY MATERIALS FIGURES AND TABLES




