# Supplementary material for: Consensus of gene expression phenotypes and prognostic risk predictors in primary lung adenocarcinoma
Source: Oncotarget. 2016 Jul 16;7(33):52957–73. doi: 10.18632/oncotarget.10641 (PMC5288161; doi:10.18632/oncotarget.10641)
Supplement: Supplementary file 1 [file oncotarget-07-52957-s001.pdf]

Consensus of gene expression phenotypes and prognostic risk predictors in primary lung adenocarcinoma

Supplementary Material

For all analyses the R statistical language was used [1].

1. Survival analysis

For survival analyses, endpoints were selected as overall survival (OS) or distant metastasis-free survival (DMFS) with a five-year censoring due to considerable differences in follow-up time between cohorts. As differences exist between cohorts regarding labeling of patient outcome other than overall survival (and the definitions are not always consistent and explicit), patient outcome stated as relapse-free survival, distant metastasis-free survival, or recurrence-free survival were interpreted as DMFS in survival analyses.

2. Gene expression data normalization

In the present study we used 17 previously reported lung cancer gene expression cohorts. 11 cohorts were analyzed using Affymetrix gene expression arrays, three cohorts using Agilent gene expression arrays, two cohorts using Illumina bead arrays, and one cohort was analyzed using RNA sequencing (The Cancer Genome Atlas, TCGA cohort). Details are listed in Supplementary Table S1.

Affymetrix data were normalized using the GC Robust Multi-array Average (GCRMA) method as described previously [2, 3], for each cohort using all cases in the cohort. Specifically, we used the just.gcrma function in the gcrma R package with default settings. For non-Affymetrix cohorts, data was obtained from public repositories, such as Gene Expression Omnibus [4], in the form of already normalized expression levels. For the TCGA [5] cohort, we used available data from 435 patients collected and processed as described elsewhere [2].

Normalized gene expression data was log2 transformed and used as measurements of gene expression in downstream analyses. For subsequent analyses, only samples annotated as adenocarcinomas were used.

3. Gene signatures

We analyzed the classification overlap between 18 adenocarcinoma gene signatures across the 17 cohorts (Table 2). In addition, we also classified samples according to a breast cancer derived proliferation signature. Of the 18 adenocarcinoma signatures, 11 signatures represented different gene expression phenotypes (GEPs) and seven signatures represented different prognostic risk prediction (RP) signatures. Of the 11 GEP signatures, four signatures were available as centroid-based classifiers (Table 2). Of the remaining seven GEP signatures a single sample predictor was not available from the original study. For these signatures, four were implemented using consensus clustering of reported gene lists, two using k-means clustering of gene lists specific for signature subtypes, and one as described in the original study (Fukui et al. [6]). Classification details for individual GEP signatures are listed below.

RP signatures were implemented as described in original studies (see below for further details).

For the different GEP and RP signatures we list below whether classifications are available from the original studies in any of the cohorts included in the current study. All classifications are available in Supplementary Table S1.

GEP - Centroids:
1. Wilkerson et al. [7]: 100% agreement with reported classifications in the Wilkerson et al. cohort [7], and the adenocarcinoma TCGA study [8] as described by Ringner et al. [9].
2. Staaf et al. [10]: No reported classifications are available using the same implementation as for Wilkerson et al. [7].
3. Planck et al. [11]: No reported classifications are available using the same implementation as for Wilkerson et al. [7].

GEP - Gene signatures:
4. Takeuchi et al. [12]: No reported sample classifications are available from the original study in any of the current cohorts.
5. Cheung et al. [13]: No explicit sample classifications are available from the original study in any of the current cohorts. However, see below in the specific section for the predictor for detailed information / comparison DO CLUSTERING?! As Cheung supp.
6. Shibata et al. [14]: No reported sample classifications are available from the original study in any of the current cohorts.
7. Fukui et al. [6]: No reported sample classifications are available from the original study in any of the current cohorts. See comment in the specific section for the predictor below.
8. Park et al. [15]: No explicit sample classifications are available from the original study in any of the current cohorts.
9: Garber et al. [16]: No explicit sample classifications are available from the original study in any of the current cohorts.
10. Bhattacharjee et al. [17]: No explicit sample classifications are available from the original study in any of the current cohorts.

Risk predictors:
11. Shedden et al., method A [18]: No explicit sample classifications are available from the original study.
12. Tang et al. [19]: The author supplied SWEAVE document was used to train and predict similar to Tang et al. No explicit sample classifications reported.
13. Okayama et al. [20]: Classifications available for XX stage I tumors. See below in the specific section for the predictor for detailed comparison. 2X2 contingency table?
14. Sun et al. [21]: No explicit sample classifications are available from the original study in current cohorts.
15. Zhu et al. [22]: No explicit sample classifications are available from the original study in current cohorts. However, see below in the specific section for the predictor for detailed information / comparison.
16. Lau et al. [23]: No explicit sample classifications are available from the original study in current cohorts.
17. Hsu et al. [24]: No explicit sample classifications are available from the original study in current cohorts. However, see below in the specific section for the predictor for detailed information / comparison.

3.1 Gene expression phenotype classification

This section lists the different methods used to classify the analyzed cohorts according to different GEP signatures.

Centroid classification

All classifications were performed on a per cohort basis. We used the classification procedure as recently validated for the Wilkerson et al. [7] GEP signature by Ringner et al. [9] for all centroid-based signatures (see Table 2). The procedure included:

1. Usage of all matching genes to the gene centroids (gene symbol match).
2. Median gene centering of normalized expression data across all samples in a cohort.
3. If multiple probe sets matched a gene in the centroids, the mean value was taken as a representation of the expression of the gene in a specific sample.
4. Pearson correlation as similarity metric
5. Gene expression subtype was called as the centroid with the highest correlation, assigning a subtype to all samples.

Fukui et al. classification

Classification according to the Fukui et al. [6] signature was performed as described in the original study on a per cohort basis. Briefly:

1. Gene expression data were median centered across all samples in a cohort.
2. Fukui signature genes were matched to expression data. If the cohort to classify was analyzed by Affymetrix U133 or U133 2 plus arrays we used the available probe set identifiers from Fukui et al., otherwise matching was based on gene identifier (gene symbol).
3. The IBC score (see Fukui et al.) was calculated for each sample.
4. Samples were divided into groups as described in Fukui et al.

Fukui et al used the 58 adenocarcinomas in Bild et al. as validation cohort, however no predictions are publicly available. Moreover, the survival analysis for this validation cohort appears to be based on 41 of the 58 cases.

GEP classification based on consensus clustering

For six of the investigated GEPs only gene signature lists were available (see Table 2). In addition, while Park et al. [15] derived a classifier, is not readily available. Consequently, seven GEP signatures needed to be implemented. The majority of these signatures were originally derived by unsupervised analysis (typically hierarchical clustering), with only gene lists available from the original studies. For four of these signatures (Park et al., Shibata et al., Takeuchi et al., and Cheung et al.) we used the signature gene lists and classified samples by consensus clustering using the ConsensusClusterPlus package [25] on a per cohort basis. When detailed cluster parameters were available in original studies we used these as parameters in the clustering. If we could not identify specific clustering details, such as linkage and similarity type, consensus clustering was performed using Pearson correlation and Ward linkage. We used the defined number of clusters from the original studies as the number of clusters to extract. Prior to clustering, gene expression data were median-centered across all samples. Matching of gene signature lists to expression data was made by gene identifier (gene symbol). If multiple probes matched for a gene the average value was used to represent the gene in the expression data. Specific ConsensusClusterPlus parameters were:

1. 2000 iterations

2. pItem=0.7, pFeature=0.7 (resampling fractions of genes and samples)

In addition, for each of the GEP gene signatures used in the consensus clustering we also calculated a metagene score, as described below in the Metagene section, considering the direction of expression, if specified. The metagene scores were used to assign a GEP class name matching the original studies to the extracted consensus clusters for each signature.

Garber et al. classification

To assign samples to the three Garber et al. [16] GEPs (AC1, AC2, AC3) we first calculated metagene scores for each sample on a per cohort basis, based on the genes reported to be characteristic for respective subtype (three such gene lists were reported by Garber et al.). First, gene expression data were median-centered across all samples in a cohort. A metagene score was calculated for each sample and subtype gene list as the average expression of all matching probes/gene identifiers. If several probe identifiers matched a gene in a specific subtype gene list, the probe set with the highest log2ratio standard deviation was selected to represent the gene in question (consistent with other metagene calculations in this study). Next, for each cohort we Z-transformed metagene scores and collected the values for all cases (n=2395) into a final matrix with 3 columns (Z-transformed metagene scores for respective subtype) and 2395 rows (samples). To assign samples to a subtype we performed k-means clustering using this matrix. Parameters were: number of centers=3, nstart=25, and iter.max=100 as parameters. To connect k-means clusters with a Garber et al. subtype label (AC1, AC2, AC3) we investigated the metagene scores for respective cluster and assigned labels to the clusters based on the patterns of metagene expression (to be in concordance with the patterns reported by Garber et al.). For instance, high expression of the AC3 metagene for a k-means cluster indicates that this cluster likely represents the AC3 subtype.

Bhattacharjee et al. classification

To assign samples to the four Bhattacharjee et al. [17] GEPs (C1, C2, C3, C4) we first calculated metagene scores for each sample on a per cohort basis, based on the genes reported to be characteristic for respective subtype (four such gene lists were reported by Bhattacharjee et al.). First, gene expression data were median-centered across all samples in a cohort. A metagene score was calculated for each sample and subtype gene list as the average expression of all matching probes/gene identifiers. If several probe identifiers matched a gene in a specific subtype gene list, the probe set with the highest log2ratio standard deviation was selected to represent the gene in question (consistent with other metagene calculations in this study). Next, for each cohort we Z-transformed metagene scores and collected values for all cases (2395) into a final matrix with 4 columns (Z-transformed metagene scores for respective subtype) and 2395 rows (samples). To assign samples to a subtype we performed k-means clustering using this matrix. Parameters were: number of centers=4, nstart=25, and iter.max=100. To connect k-means clusters with a Bhattacharjee et al. subtype labels (C1, C2, C3, C4) we investigated the metagene scores for respective cluster and assigned labels to the clusters based on the patterns of metagene expression (to be in concordance with the patterns reported by Bhattacharjee et al.). For instance, high expression of the C2 metagene for a k-means cluster indicates that this cluster likely represents the C2 subtype.

GOBO breast cancer proliferation classification

To demonstrate the association of proliferation with GEP classifications in lung cancer we created a breast cancer proliferation signature using the co-expressed genes module in GOBO [26]. By using *Ki67* (*MKI67*) as a proliferation seed gene we identified 155 up- and down-regulated genes with a standard deviation >0.7 across the 1881 breast cancer samples and an absolute correlation >0.4 to *MKI67* and at least 3 other genes using the approach described by Fredlund et al. [27]. We next classified samples according to this signature through similar consensus clustering as described for the lung cancer specific signatures.

3.2 Risk prediction classification

This section lists the different methods used to classify the analyzed cohorts according to different RP signatures.

Shedden et al. Method A classification

To assign samples according to the Shedden et al. [18] method A, which was reported as one of the best working risk stratifier models in that study, we calculated for each cohort and sample a risk score as described. When possible, Affymetrix probe set identifiers were used to match signature genes, otherwise the gene identifier (gene symbol) was used. Secondly, for each cohort we divided samples into three groups using quantiles representing low-risk, moderate risk, or high-risk groups.

Okayama 4-gene signature

For the Okayama 4-gene risk predictor, a risk score was calculated based on the information in Okayama et al. [20] on a per cohort basis. Briefly, gene expression data were median gene-centered across all samples in a cohort. We used the probe sets / probes specified by Okayama et al. for the 4 genes for different platforms whenever possible, otherwise we used the gene identifier (gene symbol) to define matching probes. If multiple probes existed for a signature gene their average expression was used. The final risk score was calculated using the equation provided by Okayama et al. and patients were stratified into three groups representing low-risk, moderate risk, or high-risk groups using quantiles, similar to Okayama et al.

For 310 patients directly mappable by provided sample identifiers in the Okayama et al. supplementary data to the current study we find a Pearson correlation of 0.684 between our scores and the reported scores from Okayama et al.

Hsu 4-gene signature

For the Hsu et al. 4-gene risk predictor, a risk score was calculated based on an implementation described by Hsu et al. [24] on a per cohort basis. Briefly, log2 transformed gene expression data for each probe in a cohort was used (no gene-centering was applied as it was considered unclear if this was performed in the original study). We used the probe sets / probes specified by Hsu et al. for the 4 genes for different platforms whenever possible, otherwise we used the gene identifier (gene symbol) to define matching probes. For the Beer et al. [28] cohort no signature genes could be matched. If multiple probes existed for a signature gene their average expression was used. Weighting for matching genes was performed by their cox regression coefficients (available from Hsu et al.) similar to Hsu et al. A risk score was next calculated as a linear combination of the weighted expression of the signature genes. Patients were classified as high-risk or low-risk based on the median score, similar to Hsu et al.

In the original study, Hsu et al. classified two cohorts, the complete NSCLC Bild et al. [29] cohort (GSE3141) and adenocarcinoma samples from the Shedden et al. cohort. To verify our implementation, we first classified the entire 111-sample NSCLC Bild et al. cohort as described above. We obtained a median risk score in the cohort of 3.65 with a range of 2.3-6.22. The corresponding values from Hsu et al. were median= 3.84 and range = 2.5- 6.68. For survival using the full overall survival follow-up, we obtained a log-rank p-value of 0.001 (rounded to 3-digits) similar as for Hsu et al (p=0.001). Moreover, in comparison to the supplementary data from Hsu et al., we find that our division into low- and high-risk gives identical number of patients with adenocarcinoma or squamous cell carcinoma in the two groups as in the original study. Taken together, our implementation seemed to generate identical results on a patient classification level as in the original study.

However, in the Shedden et al. cohort we could not obtain survival results consistent with the original study using the same classification approach. Specifically, we observed a non-significant log-rank p-value, and in the univariate analysis even a slightly better outcome (overall survival) for the high-risk group. The same pattern was observed when modifying the prediction model to e.g. include z-score transformation as an interpretation of "normal score transformation" (Hsu et al.), or when performing classification individually for the subcohorts in Shedden et al. (as performed in Hsu et al.). For the latter, when using the same classification procedure as in Bild et al. we found a median risk score of 3.69 with a range of 1.9-6.5 in the Shedden et al. cohort. To further investigate this discrepancy we plotted the expression of the individual genes for the classifications subgroups. For *ANKRD49*, expression was higher in the low-risk group (Wilcoxon's test p<1e-05) consistent with its "protective nature" (Hsu et al.), while for *RABAC1* and *EGLN2* expression was higher in the high-risk group (p<1e-05, and p<1e-05, respectively) consistent with their "risk nature". No difference between groups was observed for *LPHN1*.

Sun 50-gene signature

For the Sun et al. 50-gene risk predictor, a risk score was calculated based on an implementation as described by Sun et al. [21] on a per cohort basis. Briefly, log2 transformed gene expression data for each probe in a cohort were used (no gene-centering was applied as it was considered unclear if this was performed in the original study). Probe sets for signature genes were identified by matching to the gene identifier (gene symbol). When multiple probe identifiers existed for a signature gene their average expression was used. Weighting for matching genes was performed by their cox regression coefficients (available from Sun et al.) similar to Sun et al. A risk score was next calculated as a linear combination of the weighted expression of the signature genes. Patients were classified as high-risk or low risk similar to Sun et al. using the 60th percentile (high-risk $\geq 60^{th}$ percentile).

Zhu 15-gene signature

For the Zhu et al. 15-gene risk predictor, a risk score was calculated based on an implementation described by Zhu et al. and Der et al. [22, 30] on a per cohort basis. Briefly, gene expression data were mean gene-centered across all samples in a cohort. Whenever possible we used the probe sets specified by Zhu et al. for the 15 signature genes for different platforms, otherwise we used the gene identifier (gene symbol) to define matching probes. If multiple probes existed for a signature gene their average expression was used. In the calculation of the four principal components (PCs) for each sample we used the original rotation matrix available in the Zhu et al. data

supplement, as also used by Der et al. The final risk score was calculated using the equation provided by Zhu et al. Patients were classified as high-risk or low risk similar to Zhu et al., using the median score as cut-off. To assure that the signs of the principal component rotation matrix did not play a role for the group assignment, the high-risk group was verified to have higher expression of a proliferation metagene, otherwise the group assignments were inverted.

We confirmed our classification in three cohorts analyzed Zhu et al. or Der et al. [22, 30]. Specifically, we looked at the complete Roepman et al. [31] cohort (n=172 NSCLC, overall survival, 5-year censoring), the complete Der et al. (n=181 NSCLC, overall survival, 5 year censoring) and the complete JBR.10 [22] cohort (n=133 NSCLC, disease specific survival). Although we could not reproduce all outcome p-values or identical group sizes (high- and low-risk) to those in the original study, we observed significant agreement in the cohorts for outcome compared with the original studies, including the observation arm in the JBR.10 study (Zhu et al, Figure 1), the Der et al. total cohort and adenocarcinoma subgroup specifically (Der et al., Figures 1 and 2), and the total Roepman et al. cohort (Resembling Figure 2 in Zhu et al.). We obtained the best agreement when using mean instead of median gene-centering.

Tang 18-gene signature

For the Tang et al. 18-gene risk predictor, a risk score was calculated based on an implementation as described by Tang et al. [19] on a per cohort basis. We used the 18-gene signature from Tang et al. as this signature was most evaluated for prognostic significance in that study. Matching of probe identifiers to signature genes was made using gene identifiers (gene symbol). Prior to classification, gene expression data were median gene-centered for each probe identifier across samples in a cohort. We trained a classifier for the 18 genes in the Shedden et al. cohort using 442 cases with outcome data (overall survival) based on the R Sweave documentation provided by Tang et al. We applied the predictor to remaining cohorts by first matching the number of available signature genes to the trained model and the test cohort (some platforms did not include all signature genes). In the superpc.predict function we used the same values for the pcthr and number of components as in the Tang et al. Sweave document. The risk score was taken as the $v.pred.1df value from the fitted object similar to Tang et al. Patients were classified as high-risk or low-risk based on the median score similar to Tang et al.

Lau 3-gene signature

For the Lau et al. 3-gene risk predictor, a risk score was calculated based on an implementation as described by Lau et al. [23] on a per cohort basis. Briefly, gene expression data were median gene-centered for each probe identifier across all samples in a cohort. For the three genes we used the probe sets / probes specified by either Lau et al. or probe sets / probes specified by other signatures that also included these genes. If no specific probe identifiers could be matched, we used the gene identifier (gene symbol) to define matching probes. If multiple probes existed for a signature gene their average expression was used. The final risk score was calculated using the equation provided by Lau et al. Patients were classified as high-risk or low risk similar to Lau et al. based on their risk score. In some cohorts, e.g. Roepman et al., all three genes could not be identified. For these cohorts classification was still performed according to the rules by Lau et al. for remaining genes (at least two genes).

4. Gene expression metagenes

Five metagenes representing different biological processes: proliferation, immune response, stroma/extracellular matrix, Napsin A / surfactant expression, and basal-squamous expression, identified from lung cancer gene expression data were obtained from Karlsson et al. [32]. If several probe identifiers matched a gene in a specific metagene, the probe set with the highest log2ratio standard deviation was selected to represent the gene in question. A metagene score was calculated for each sample on a per cohort basis, based on normalized and median centered gene expression data (across all samples in a cohort) as the average expression of all matching probes/gene identifiers.

Comparison of GEP signature metagenes or risk scores from RP models with the five "biological" metagenes was made by first performing a Z-scale transformation of the metagene/risk scores for samples in each cohort respectively. The Z-scores for all cases were then collected and comparisons between different metagenes were made. Linear regression was calculated and the standard R^2 metric was extracted as a measurement of the fit using the standard lm function in R.

For the cell cycle progression (CCP) [33] and CIN70 signature [34] we derived proliferation scores similar to the breast cancer proliferation signature, by taking the average expression of included genes per sample, on a per cohort basis.

5. Definition of consensus sample groups

To identify consensus groups of samples across 10 GEP classifiers (excluding the AC1/AC2 classifier by Staaf et al. [10]) we established a set of subtype classification rules for three subgroups (CONSENSUS_1, CONSENSUS_2, CONSENSUS_3) based on the subtype characteristics from the original studies (mainly definition of low- and high-risk groups). The selection of three consensus groups implies that two-group classifiers will be divided into additional subgroups. This division will mainly affect the high-risk outcome group for these signatures in order to match the mapping to the low-risk groups defined by Garber et al., Bhattacharjee et al. and Wilkerson et al. Next, we identified samples with consensus overlap for ≥8 classifiers. Groups were defined as:

CONSENSUS_1:

Wilkerson et al. :	TRU-type (class)
Garber et al. :	AC1 (class)
Bhattacharjee et al. :	C4 (class)
Planck et al. EGFR :	CCL1 (class)
Planck et al. EGFRwt/KRASwt :	CCL2 (class)
Fukui et al. :	BC-low (class)
Park et al. :	S_C2 (class)
Cheung et al. :	Alveolar (class)
Shibata et al. :	Alveolar (class)
Takeuchi et al. :	TRU (class)

CONSENSUS_2:

Wilkerson et al. :	Proximal-proliferative (class)
Garber et al. :	AC2 (class)
Bhattacharjee et al. :	C2 (class)
Planck et al. EGFR :	CCL2 (class)
Planck et al. EGFRwt/KRASwt :	CCL1 (class)
Fukui et al. :	BC-high (class)

Park et al. : F_C1 (class)
Cheung et al. : DASC (class)
Shibata et al. : Bronchial (class)
Takeuchi et al. : non-TRU (class)

CONSENSUS_3:

Wilkerson et al. : Proximal-inflammatory (class)
Garber et al. : AC3 (class)
Bhattacharjee et al. : C1 (class)
Planck et al. EGFR : CCL2 (class)
Planck et al. EGFRwt/KRASwt : CCL1 (class)
Fukui et al. : BC-high (class)
Park et al. : F_C1 (class)
Cheung et al. : DASC (class)
Shibata et al. : Bronchial (class)
Takeuchi et al. : non-TRU (class)

References

1. The R Project for Statistical Computing.
2. Karlsson A, Ringner M, Lauss M, Botling J, Micke P, Planck M and Staaf J. Genomic and transcriptional alterations in lung adenocarcinoma in relation to smoking history. Clin Cancer Res. 2014; 20(18):4912-4924.
3. Planck M, Edlund K, Botling J, Micke P, Isaksson S and Staaf J. Genomic and transcriptional alterations in lung adenocarcinoma in relation to EGFR and KRAS mutation status. PLoS ONE. 2013; 8(10):e78614.
4. Gene Expression Omnibus.
5. The Cancer Genome Atlas.
6. Fukui T, Shaykhiev R, Agosto-Perez F, Mezey JG, Downey RJ, Travis WD and Crystal RG. Lung adenocarcinoma subtypes based on expression of human airway basal cell genes. The European respiratory journal. 2013; 42(5):1332-1344.
7. Wilkerson MD, Yin X, Walter V, Zhao N, Cabanski CR, Hayward MC, Miller CR, Socinski MA, Parsons AM, Thorne LB, Haithcock BE, Veeramachaneni NK, Funkhouser WK, et al. Differential pathogenesis of lung adenocarcinoma subtypes involving sequence mutations, copy number, chromosomal instability, and methylation. PLoS ONE. 2012; 7(5):e36530.
8. The Cancer Genome Atlas Network A. Comprehensive molecular profiling of lung adenocarcinoma. Nature. 2014; 511(7511):543-550.
9. Ringner M, Jonsson G and Staaf J. Prognostic and chemotherapy predictive value of gene expression phenotypes in primary lung adenocarcinoma. Clin Cancer Res. 2015; 22(1):218-229.
10. Staaf J, Jonsson G, Jonsson M, Karlsson A, Isaksson S, Salomonsson A, Pettersson HM, Soller M, Ewers SB, Johansson L, Jonsson P and Planck M. Relation between smoking history and gene expression profiles in lung adenocarcinomas. BMC Med Genomics. 2012; 5:22.
11. Planck M, Isaksson S, Veerla S and Staaf J. Identification of transcriptional subgroups in EGFR-mutated and EGFR/KRAS-wild type lung adenocarcinoma

reveals gene signatures associated with patient outcome. Clin Cancer Res. 2013; 19(18):5116-5126.
12. Takeuchi T, Tomida S, Yatabe Y, Kosaka T, Osada H, Yanagisawa K, Mitsudomi T and Takahashi T. Expression profile-defined classification of lung adenocarcinoma shows close relationship with underlying major genetic changes and clinicopathologic behaviors. J Clin Oncol. 2006; 24(11):1679-1688.
13. Cheung WK, Zhao M, Liu Z, Stevens LE, Cao PD, Fang JE, Westbrook TF and Nguyen DX. Control of alveolar differentiation by the lineage transcription factors GATA6 and HOPX inhibits lung adenocarcinoma metastasis. Cancer cell. 2013; 23(6):725-738.
14. Shibata T, Hanada S, Kokubu A, Matsuno Y, Asamura H, Ohta T, Sakamoto M and Hirohashi S. Gene expression profiling of epidermal growth factor receptor/KRAS pathway activation in lung adenocarcinoma. Cancer Sci. 2007; 98(7):985-991.
15. Park YY, Park ES, Kim SB, Kim SC, Sohn BH, Chu IS, Jeong W, Mills GB, Byers LA and Lee JS. Development and validation of a prognostic gene-expression signature for lung adenocarcinoma. PLoS ONE. 2012; 7(9):e44225.
16. Garber ME, Troyanskaya OG, Schluens K, Petersen S, Thaesler Z, Pacyna-Gengelbach M, van de Rijn M, Rosen GD, Perou CM, Whyte RI, Altman RB, Brown PO, Botstein D, et al. Diversity of gene expression in adenocarcinoma of the lung. Proceedings of the National Academy of Sciences of the United States of America. 2001; 98(24):13784-13789.
17. Bhattacharjee A, Richards WG, Staunton J, Li C, Monti S, Vasa P, Ladd C, Beheshti J, Bueno R, Gillette M, Loda M, Weber G, Mark EJ, et al. Classification of human lung carcinomas by mRNA expression profiling reveals distinct adenocarcinoma subclasses. Proceedings of the National Academy of Sciences of the United States of America. 2001; 98(24):13790-13795.
18. Shedden K, Taylor JM, Enkemann SA, Tsao MS, Yeatman TJ, Gerald WL, Eschrich S, Jurisica I, Giordano TJ, Misek DE, Chang AC, Zhu CQ, Strumpf D, et al. Gene expression-based survival prediction in lung adenocarcinoma: a multi-site, blinded validation study. Nature medicine. 2008; 14(8):822-827.
19. Tang H, Xiao G, Behrens C, Schiller J, Allen J, Chow CW, Suraokar M, Corvalan A, Mao JH, White M, Wistuba, II, Minna JD and Xie Y. A 12-gene set predicts survival benefits from adjuvant chemotherapy in non-small-cell lung cancer patients. Clin Cancer Res. 2013; 19(6):1577-1586.
20. Okayama H, Schetter AJ, Ishigame T, Robles AI, Kohno T, Yokota J, Takenoshita S and Harris CC. The expression of four genes as a prognostic classifier for stage I lung adenocarcinoma in 12 independent cohorts. Cancer Epidemiol Biomarkers Prev. 2014; 23(12):2884-2894.
21. Sun Z, Wigle DA and Yang P. Non-overlapping and non-cell-type-specific gene expression signatures predict lung cancer survival. J Clin Oncol. 2008; 26(6):877-883.
22. Zhu CQ, Ding K, Strumpf D, Weir BA, Meyerson M, Pennell N, Thomas RK, Naoki K, Ladd-Acosta C, Liu N, Pintilie M, Der S, Seymour L, et al. Prognostic and predictive gene signature for adjuvant chemotherapy in resected non-small-cell lung cancer. J Clin Oncol. 2010; 28(29):4417-4424.
23. Lau SK, Boutros PC, Pintilie M, Blackhall FH, Zhu CQ, Strumpf D, Johnston MR, Darling G, Keshavjee S, Waddell TK, Liu N, Lau D, Penn LZ, et al. Three-gene

prognostic classifier for early-stage non small-cell lung cancer. J Clin Oncol. 2007; 25(35):5562-5569.
24. Hsu YC, Yuan S, Chen HY, Yu SL, Liu CH, Hsu PY, Wu G, Lin CH, Chang GC, Li KC and Yang PC. A four-gene signature from NCI-60 cell line for survival prediction in non-small cell lung cancer. Clin Cancer Res. 2009; 15(23):7309-7315.
25. Wilkerson MD and Hayes DN. ConsensusClusterPlus: a class discovery tool with confidence assessments and item tracking. Bioinformatics. 2010; 26(12):1572-1573.
26. Ringner M, Fredlund E, Hakkinen J, Borg A and Staaf J. GOBO: Gene Expression-Based Outcome for Breast Cancer Online. PLoS ONE. 2011; 6(3):e17911.
27. Fredlund E, Staaf J, Rantala JK, Kallioniemi O, Borg A and Ringner M. The gene expression landscape of breast cancer is shaped by tumor protein p53 status and epithelial-mesenchymal transition. Breast Cancer Res. 2012; 14(4):R113.
28. Beer DG, Kardia SL, Huang CC, Giordano TJ, Levin AM, Misek DE, Lin L, Chen G, Gharib TG, Thomas DG, Lizyness ML, Kuick R, Hayasaka S, et al. Gene-expression profiles predict survival of patients with lung adenocarcinoma. Nature medicine. 2002; 8(8):816-824.
29. Bild AH, Yao G, Chang JT, Wang Q, Potti A, Chasse D, Joshi MB, Harpole D, Lancaster JM, Berchuck A, Olson JA, Jr., Marks JR, Dressman HK, et al. Oncogenic pathway signatures in human cancers as a guide to targeted therapies. Nature. 2006; 439(7074):353-357.
30. Der SD, Sykes J, Pintilie M, Zhu CQ, Strumpf D, Liu N, Jurisica I, Shepherd FA and Tsao MS. Validation of a Histology-Independent Prognostic Gene Signature for Early-Stage, Non-Small-Cell Lung Cancer Including Stage IA Patients. J Thorac Oncol. 2014; 9(1):59-64.
31. Roepman P, Horlings HM, Krijgsman O, Kok M, Bueno-de-Mesquita JM, Bender R, Linn SC, Glas AM and van de Vijver MJ. Microarray-based determination of estrogen receptor, progesterone receptor, and HER2 receptor status in breast cancer. Clin Cancer Res. 2009; 15(22):7003-7011.
32. Karlsson A, Jonsson M, Lauss M, Brunnstrom H, Jonsson P, Borg A, Jonsson G, Ringner M, Planck M and Staaf J. Genome-wide DNA methylation analysis of lung carcinoma reveals one neuroendocrine and four adenocarcinoma epitypes associated with patient outcome. Clin Cancer Res. 2014; 20(23):6127-6140.
33. Cuzick J, Swanson GP, Fisher G, Brothman AR, Berney DM, Reid JE, Mesher D, Speights VO, Stankiewicz E, Foster CS, Moller H, Scardino P, Warren JD, et al. Prognostic value of an RNA expression signature derived from cell cycle proliferation genes in patients with prostate cancer: a retrospective study. The lancet oncology. 2011; 12(3):245-255.
34. Carter SL, Eklund AC, Kohane IS, Harris LN and Szallasi Z. A signature of chromosomal instability inferred from gene expression profiles predicts clinical outcome in multiple human cancers. Nature genetics. 2006; 38(9):1043-1048.

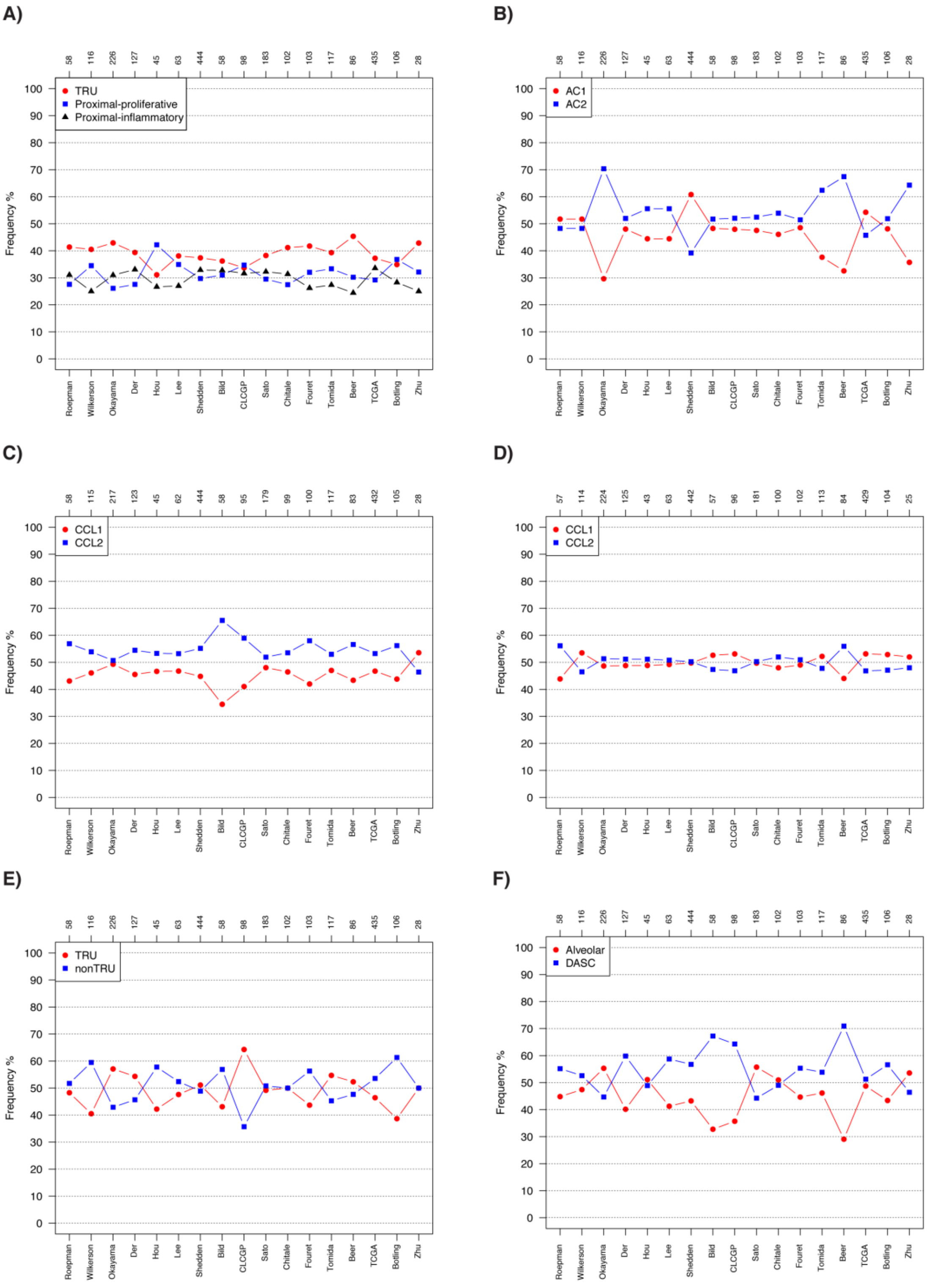
A)
TRU
Proximal-proliferative
Proximal-inflammatory
B)
AC1
AC2
C)
CCL1
CCL2
D)
CCL1
CCL2
E)
TRU
nonTRU
F)
Alveolar
DASC
Frequency %
Roepman
Wilkerson
Okayama
Der
Hou
Lee
Shedden
Bild
CLCGP
Sato
Chitale
Fouret
Tomida
Beer
TCGA
Botling
Zhu

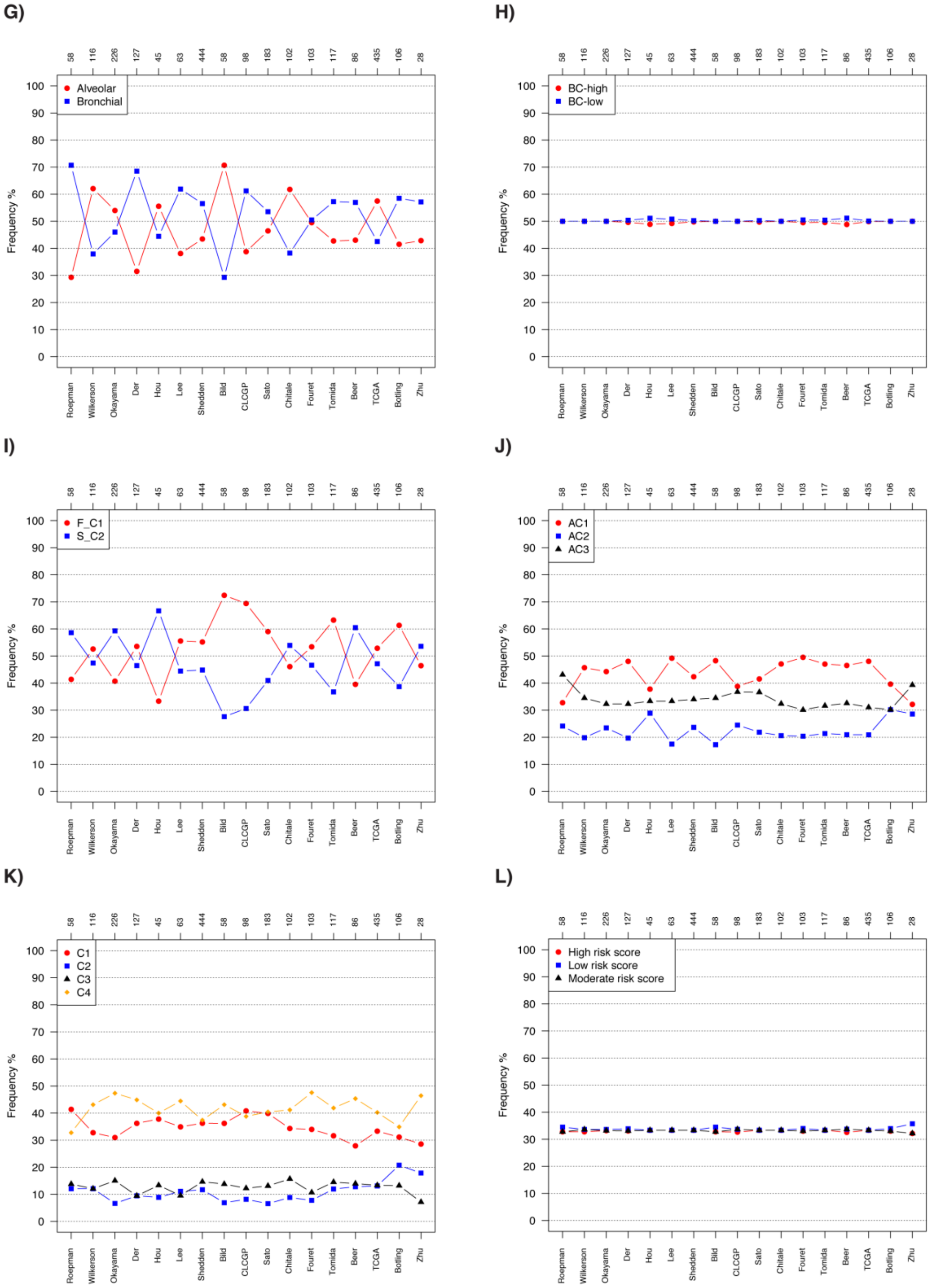

G)
Alveolar
Bronchial
H)
BC-high
BC-low
I)
F_C1
S_C2
J)
AC1
AC2
AC3
K)
C1
C2
C3
C4
L)
High risk score
Low risk score
Moderate risk score
Frequency %
58 116 226 127 45 63 444 58 98 183 102 103 117 86 435 106 28
Roepman Wilkerson Okayama Der Hou Lee Shedden Bild CLCGP Sato Chitale Fouret Tomida Beer TCGA Botling Zhu

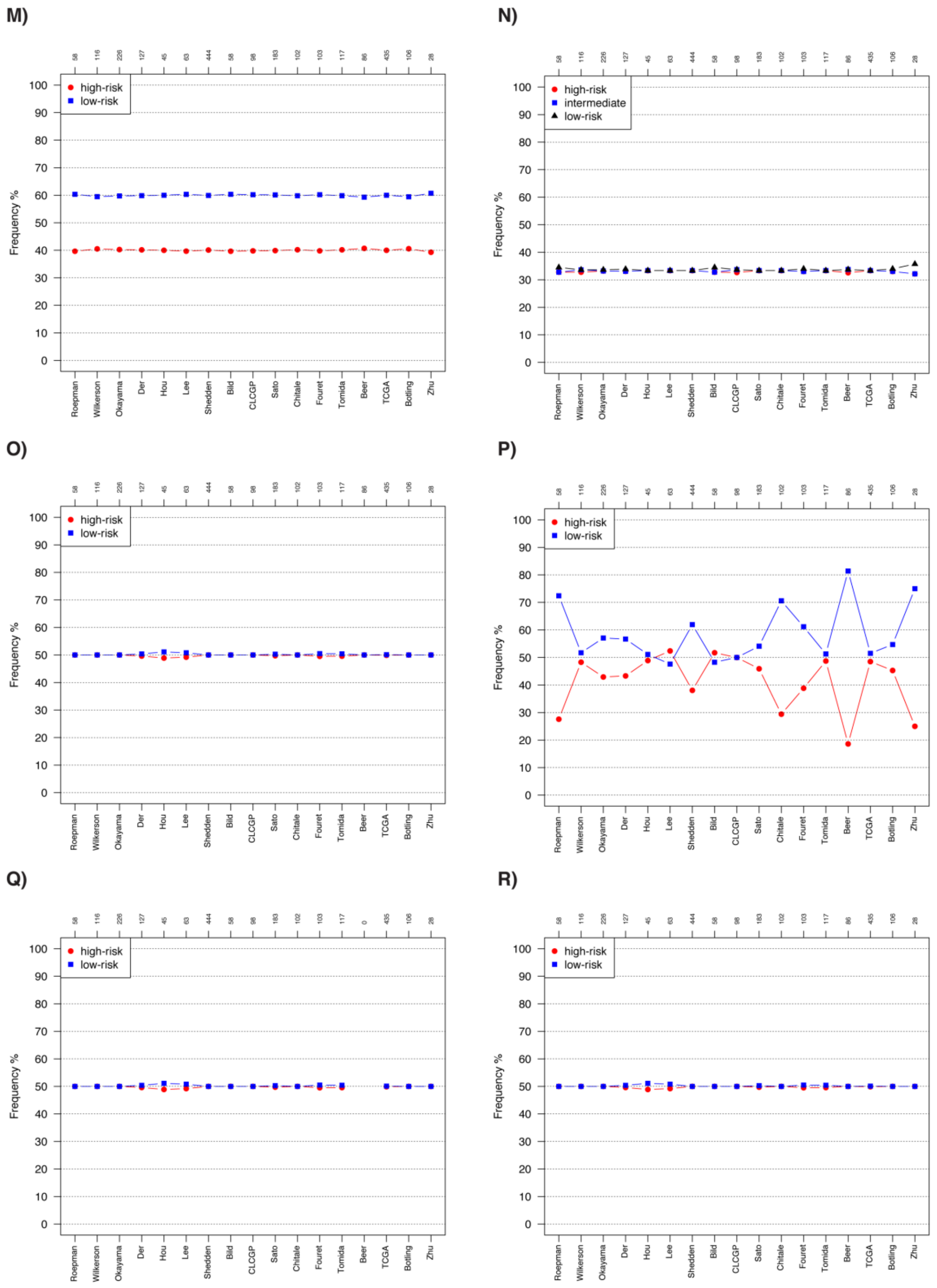

Supplementary Figure S1. Gene expression phenotype proportions for 18 lung adenocarcinoma signatures across 17 cohorts. (**A**) Wilkerson et al. (**B**) Staaf et al. (**C**) Planck et al. EGFR-centroids. (**D**) Planck et al. EGFR and KRAS-negative centroids. (**E**) Takeuchi et al. (**F**) Cheung et al. (**G**) Shibata et al. (**H**)

Fukui et al. (**I**) Park et al. (**J**) Garber et al. (**K**) Bhattacharjee et al. (**L**) Shedden et al. Method A. (**M**) Sun et al. (**N**) Okayama et al. (**O**) Zhu et al. (**P**) Lau et al. (**Q**) Hsu et al. (**R**) Tang et al.

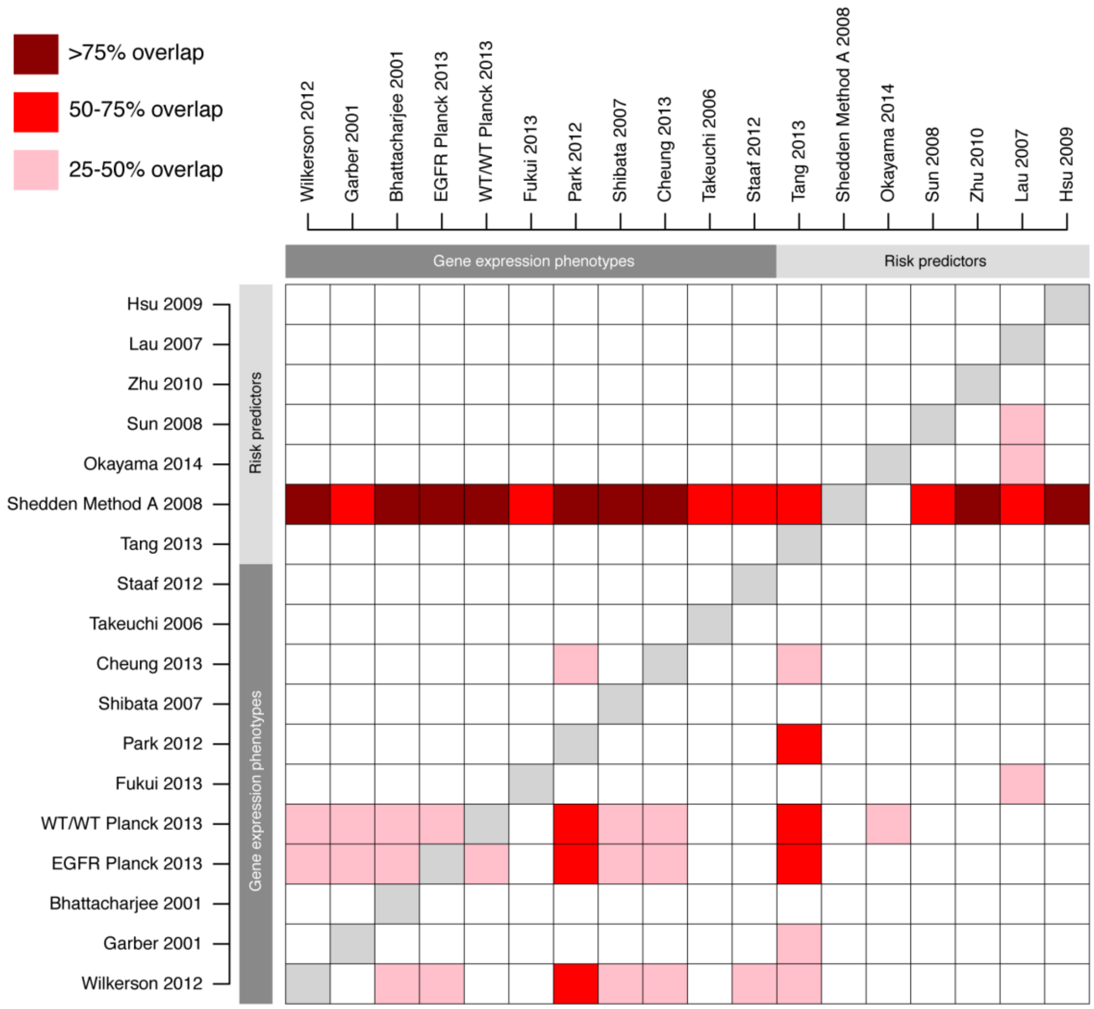

Supplementary Figure S2. Gene overlap analysis between analyzed gene expression phenotype and risk prediction signatures. Gene overlap (%) was calculated based on overlap between signature pairs for reported unique gene identifiers (gene symbol), and are displayed in % per row (denominator in calculation is the number of genes in signature for a specific row).

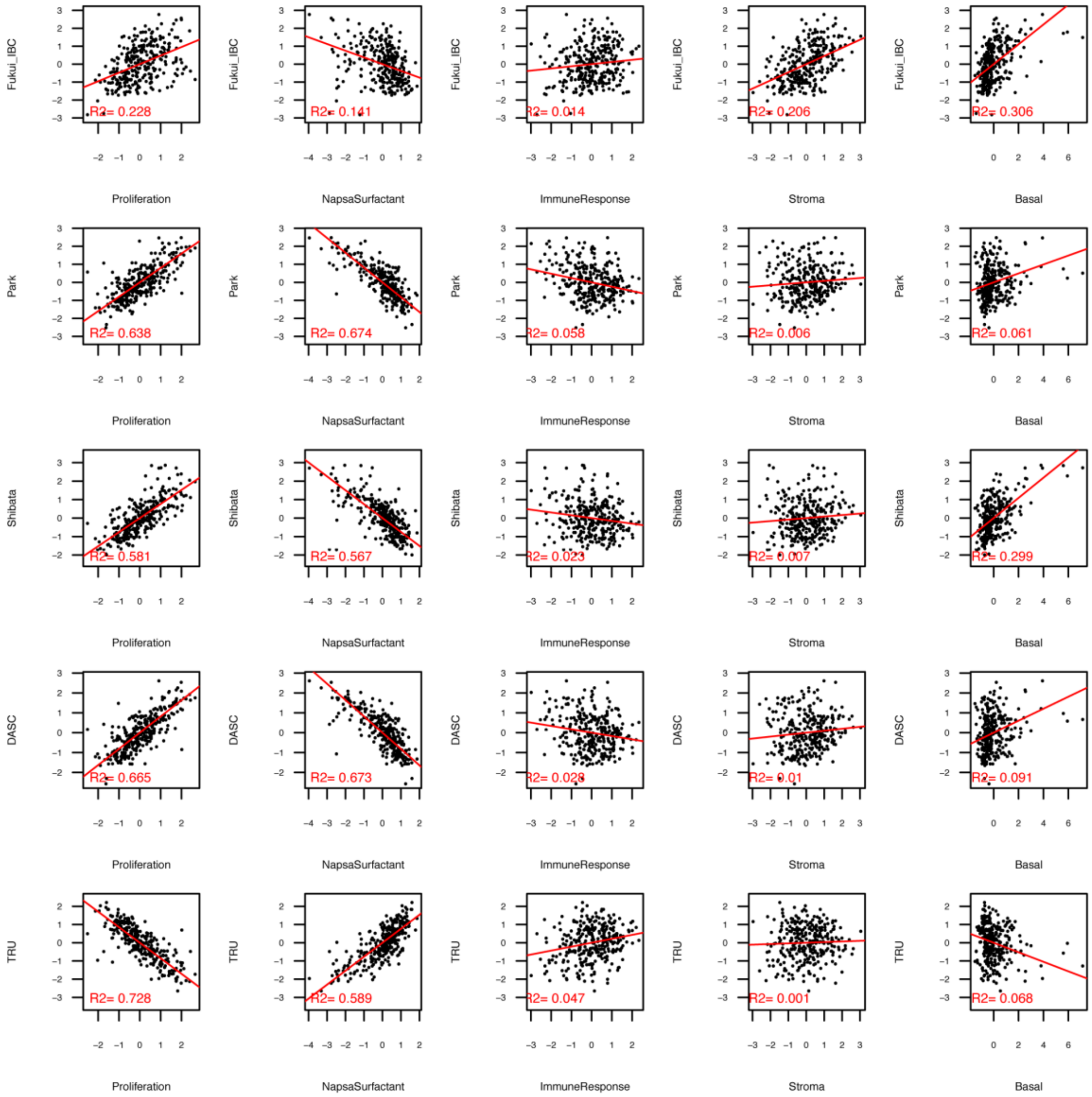

Fukui_IBC
Park
Shibata
DASC
TRU
Proliferation
NapsaSurfactant
ImmuneResponse
Stroma
Basal
R2= 0.228
R2= 0.141
R2= 0.014
R2= 0.206
R2= 0.306
R2= 0.638
R2= 0.674
R2= 0.058
R2= 0.006
R2= 0.061
R2= 0.581
R2= 0.567
R2= 0.023
R2= 0.007
R2= 0.299
R2= 0.665
R2= 0.673
R2= 0.028
R2= 0.01
R2= 0.091
R2= 0.728
R2= 0.589
R2= 0.047
R2= 0.001
R2= 0.068

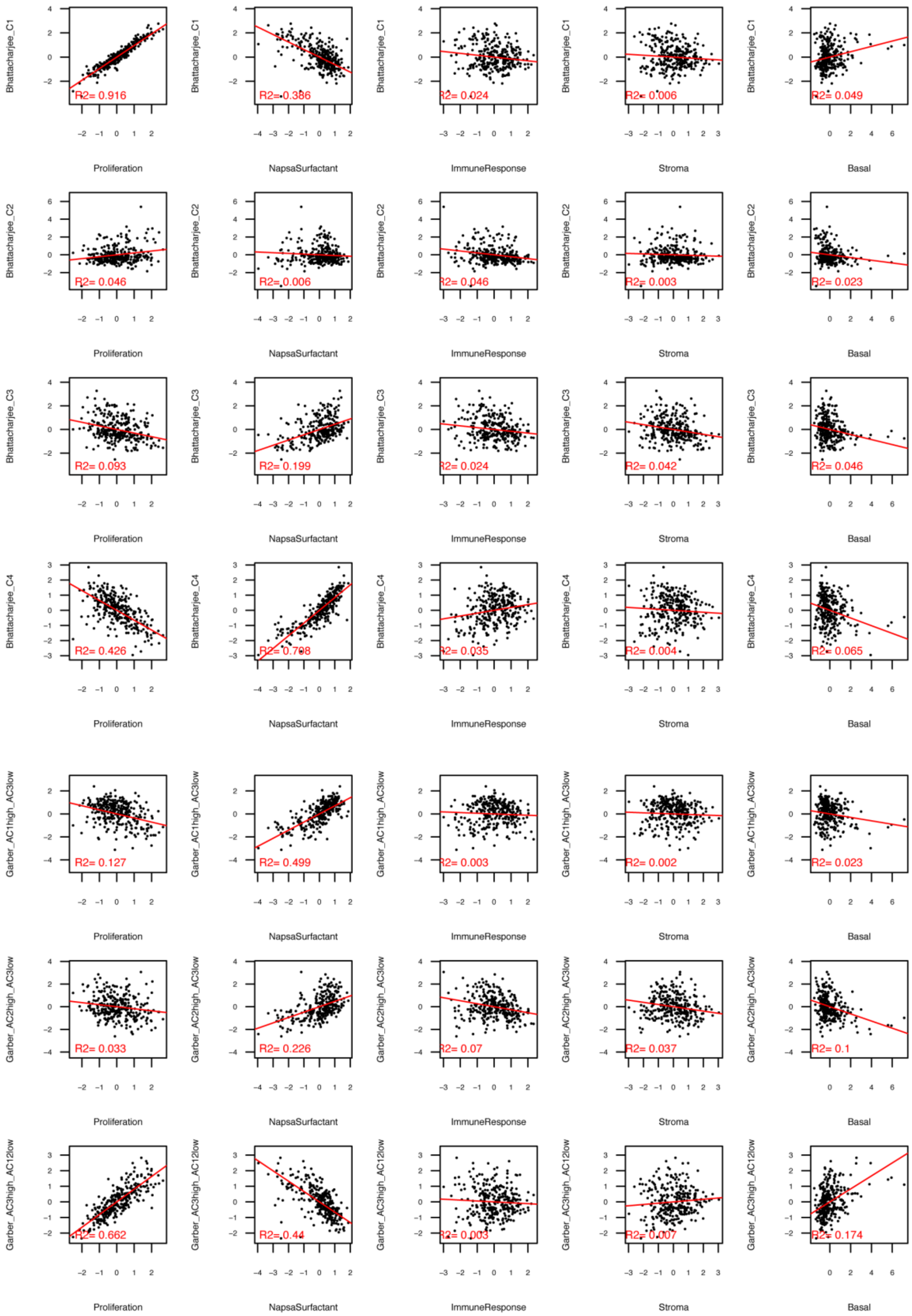
Bhattacharjee_C1
R2= 0.916
R2= 0.386
R2= 0.024
R2= 0.006
R2= 0.049
Bhattacharjee_C2
R2= 0.046
R2= 0.006
R2= 0.046
R2= 0.003
R2= 0.023
Bhattacharjee_C3
R2= 0.093
R2= 0.199
R2= 0.024
R2= 0.042
R2= 0.046
Bhattacharjee_C4
R2= 0.426
R2= 0.798
R2= 0.035
R2= 0.004
R2= 0.065
Garber_AC1high_AC3low
R2= 0.127
R2= 0.499
R2= 0.003
R2= 0.002
R2= 0.023
Garber_AC2high_AC3low
R2= 0.033
R2= 0.226
R2= 0.07
R2= 0.037
R2= 0.1
Garber_AC3high_AC12low
R2= 0.662
R2= 0.44
R2= 0.003
R2= 0.007
R2= 0.174
Proliferation
NapsaSurfactant
ImmuneResponse
Stroma
Basal

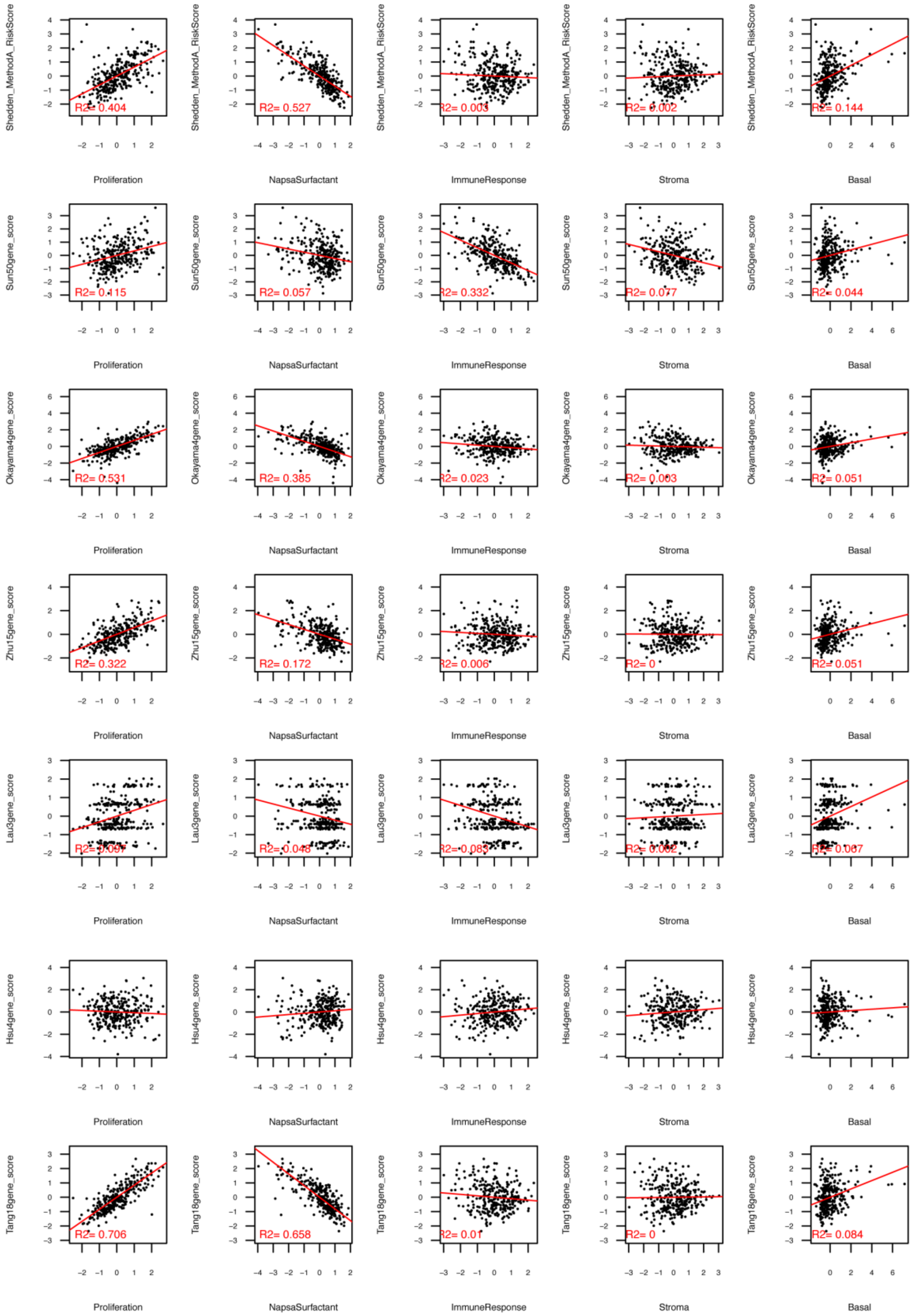

Supplementary Figure S3. Relationship between gene signatures and metagenes reflecting biological processes in lung cancer. Analysis of relationship between 14 gene signatures for different gene expression

phenotypes or risk predictors (rows) with expression of five metagenes representing biological process identified in lung cancer (columns). For the Bhattacharjee et al. and Garber et al. signatures, analyses are performed for the different signature subcomponents. Gene signature scores, risk scores, and metagene expression scores were calculated as described in Supplementary Information. For each signature / metagene / risk predictor we Z-transformed values individually for each cohort prior to pooling cohorts. Linear regression was performed to estimate the linear relationship between gene signatures and biological metagenes using the lm function in R with the R^2 value displayed. The panels represent a subplotting of every eighth data point for the purpose of size reduction, whereas the regression line represents the result of an analysis using all data points.
